# Human Remains from the Pleistocene-Holocene Transition of Southwest China Suggest a Complex Evolutionary History for East Asians

**DOI:** 10.1371/journal.pone.0031918

**Published:** 2012-03-14

**Authors:** Darren Curnoe, Ji Xueping, Andy I. R. Herries, Bai Kanning, Paul S. C. Taçon, Bao Zhende, David Fink, Zhu Yunsheng, John Hellstrom, Luo Yun, Gerasimos Cassis, Su Bing, Stephen Wroe, Hong Shi, William C. H. Parr, Huang Shengmin, Natalie Rogers

**Affiliations:** 1 School of Biological, Earth and Environmental Sciences, University of New South Wales, Sydney, New South Wales, Australia; 2 Yunnan Institute of Cultural Relics and Archeology, Kunming, Yunnan, China; 3 Archeology Research Center, Yunnan University, Kunming, Yunnan, China; 4 Archaeomagnetism Laboratory, Archaeology Program, School of Historical and European Studies, La Trobe University, Melbourne, Victoria, Australia; 5 Honghe Prefectural Institute of Cultural Relics, Mengzi, Yunnan, China; 6 Place, Evolution and Rock Art Heritage Unit, School of Humanities, Gold Coast Campus, Griffith University, Southport, Queensland, Australia; 7 Mengzi Institute of Cultural Relics, Mengzi, Yunnan, China; 8 Institute for Environmental Research, Australian Nuclear Science and Technology Organisation, Sydney, Australia; 9 School of Earth Sciences, University of Melbourne, Melbourne, Victoria, Australia; 10 State Key Laboratory of Genetic Resources and Evolution, Kunming Institute of Zoology and Kunming Primate Research Centre, Chinese Academy of Sciences, Kunming, China; 11 Youjiang Nationalities Museum, Baise, Guangxi, China; University of Florence, Italy

## Abstract

**Background:**

Later Pleistocene human evolution in East Asia remains poorly understood owing to a scarcity of well described, reliably classified and accurately dated fossils. Southwest China has been identified from genetic research as a hotspot of human diversity, containing ancient mtDNA and Y-DNA lineages, and has yielded a number of human remains thought to derive from Pleistocene deposits. We have prepared, reconstructed, described and dated a new partial skull from a consolidated sediment block collected in 1979 from the site of Longlin Cave (Guangxi Province). We also undertook new excavations at Maludong (Yunnan Province) to clarify the stratigraphy and dating of a large sample of mostly undescribed human remains from the site.

**Methodology/Principal Findings:**

We undertook a detailed comparison of cranial, including a virtual endocast for the Maludong calotte, mandibular and dental remains from these two localities. Both samples probably derive from the same population, exhibiting an unusual mixture of modern human traits, characters probably plesiomorphic for later *Homo*, and some unusual features. We dated charcoal with AMS radiocarbon dating and speleothem with the Uranium-series technique and the results show both samples to be from the Pleistocene-Holocene transition: ∼14.3-11.5 ka.

**Conclusions/Significance:**

Our analysis suggests two plausible explanations for the morphology sampled at Longlin Cave and Maludong. First, it may represent a late-surviving archaic population, perhaps paralleling the situation seen in North Africa as indicated by remains from Dar-es-Soltane and Temara, and maybe also in southern China at Zhirendong. Alternatively, East Asia may have been colonised during multiple waves during the Pleistocene, with the Longlin-Maludong morphology possibly reflecting deep population substructure in Africa prior to modern humans dispersing into Eurasia.

## Introduction

Research about the evolution of modern humans has historically focused on the fossil records of Europe and Africa as well as the Levantine corridor connecting them. As a result, the role of the vast Asian continent in this evolutionary episode remains largely unknown. Human remains from the Upper Pleistocene of South Asia are scarce, being confined to just two sites possibly within the 33-25 thousand years (or ka) range [1]. In East Asia, human fossils are more numerous [2], but their significance has been difficult to assess due to poor knowledge of their geological context and inadequate dating [1], [2]–[3]. For clarity, we consider East Asia to comprise the geographic region bordered by the Ural Mountains in the west, the Himalayan Plateau in the southwest, Bering Strait in the northeast, and extending into island southeast Asia.

One widely discussed candidate for the oldest modern human in East Asia is the skeleton from Liujiang, southern China [2]. Yet, the geological age of this individual has “been an everlasting dispute since the discovery of the fossils in 1958” as “there is no documentation on the exact stratigraphic position of the human remains” [4, p. 62]. As a result, its estimated age lies within the broad range of >153-30 ka [2], [4]. The age of the Upper Cave (Zhoukoudian) remains is similarly problematic and has been a major source of uncertainty since their discovery in the 1930s, with estimates ranging from ∼33-10 ka [2], [4]. Furthermore, the Niah Cave child from East Malaysia possesses uncertain provenience [5]. However, a recent field and lab program aiming to assess the stratigraphy and dating of the deposits at the site has proposed an age of ∼45-39 ka for this cranium [5].

Most other candidates for the earliest modern humans in East Asia are similarly problematic. Among the human remains recovered from Tabon Cave, Philippines, the only taxonomically diagnostic specimen is a frontal bone assigned to *H. sapiens* [6], and dated to 16.5±2 ka [7]. Moreover, the oldest specimen from the site – directly dated to 47+11/−10 ka [7] – might be from an orangutan [6]. At Callao Cave, Luzon, a hominin metatarsal has been directly dated to an estimated 66.7±1 ka [8]. This specimen is, however, difficult to classify reliably, making its assignment to *H. sapiens* uncertain [8]. A recently described individual from Tianyuan Cave near Zhoukoudian town, northeast China, is estimated to be ∼42-39 ka [9]. The Tianyuan partial skeleton comprises 34 pieces apparently from the same individual, its femur being directly dated to 40,328±816 cal. yr BP [9]. This specimen seems to provide the best candidate for the earliest modern human in East Asia, but is significantly younger (>20 kya) than genetic clock estimates for colonisation of the region (see below). Finally, a mandibular fragment from Zhirendong, southern China, has been dated on stratigraphic grounds to >100 ka [10]. Unfortunately, the specimen is fragmentary and possesses a mosaic of archaic and modern characters also making its taxonomic status unclear [10]–[11].

Given ongoing uncertainty surrounding the human fossil record, palaeoanthropologists have come to rely on the results of genetic sequencing of samples from living populations to reconstruct the origins of modern humans in East Asia. Genetic research suggests that the earliest humans dispersed into Eurasia from Africa around 70-60 ka, rapidly colonising Southeast Asia and Australasia after this time [12]–[15]. This seems to have been followed by a later migration within Eurasia after 40-30 ka, adding the founding lineages of modern Northeast Asians and Europeans [15]. Several later migrations seem to have occurred within the region, some associated with the Neolithic [12]–[14]. Finally, DNA extracted from a >50 ka hominin fossil from Denisova Cave in Central Asia belonging within the Neandertal lineage shares features exclusively with Aboriginal Southeast Asians and Australasians [16]–[18]. This has been interpreted as: 1) evidence for interbreeding between the ‘Denisovans’ and the earliest modern humans to colonise the region; and 2) implying occupation of Southeast Asia by this archaic population during the Upper Pleistocene [16]–[17].

**Figure 1:**
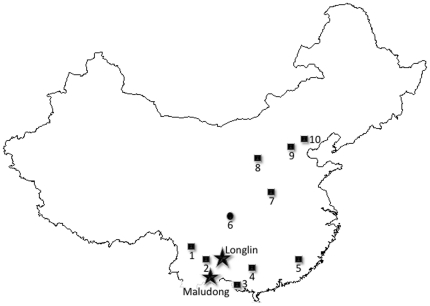
Geographic location of Longlin and Maludong in relation to major later Pleistocene human fossil sites in mainland China. 1 = Lijiang; 2 = Longtanshan; 3 = Zhirendong; 4 = Liujiang; 5 = Maba; 6 = Ziyang; 7 = Huanglong; 8 = Salawusu; 9 = Xujiayao; 10 = Zhuoukoudian-Upper Cave.

Given the central importance of the East Asian fossil record to testing regional and global scenarios of human evolution, in 2008 we began a collaborative research project with the aim of determining the age and providing detailed comparisons of possible Pleistocene human remains from southwest China. This paper focuses on human remains from two localities: Longlin Cave (Longlin or LL) and Malu Cave (Maludong or MLDG) (Figure 1).

The Longlin human remains were discovered opportunistically in 1979 by a petroleum geologist (Li Changqing) in a cave near De'e, Longlin County, Guangxi Zhuang Autonomous Region, Guangxi Province (Figure 1). A block of consolidated fine-grained sediment containing the human remains, unidentifiable animal bones, charcoal and burnt clay fragments was removed from the cave and taken to Kunming in neighbouring Yunnan Province shortly after its discovery. A partial mandible and some fragments of postcranial bone were prepared from the block at this time [2], although, the remainder of the skull and other postcranial bones were only prepared from the sediment by our team during 2010. During preparation we recovered a thin flowstone adhering to the surface of the vault of the partial LL 1 skeleton, while charcoal fragments were collected from sediment within its endocranial cavity. Association of cranium, mandible and postcranial elements with similar preservation from within a small (<1 m^3^) block of sediment suggests that post-depositional disturbance was limited. The cave has been closed to the public and we have so far been unable to undertake research to clarify the stratigraphy and geological context of the human remains.

Maludong is a partially mined cave fill located near the city of Mengzi, Honghe Prefecture, Hani and Yi Autonomous Region, southeast Yunnan Province [2], (Figure 1). The site was originally excavated in 1989 by a Chinese team including one of us (BZ), and most of the fossil and archaeological materials were recovered at that time [19]. Excavations during 2008 by several of the present authors (DC, JX, AH, BK, BZ, ZY & LY) allowed for a re-evaluation of the remaining stratigraphic section and the collection of a large number of samples for dating and archaeomagnetic analysis. Additional human remains were discovered during the current study, both during our small-scale excavation (50×50×370 cm) for stratigraphic analysis and from unstudied and unsorted fossil collections recovered during the 1989 field season.

## Results

### Dating analyses

Radiocarbon dating of charcoal from sediment removed from within the endocranial cavity of LL 1 provided an age of 11,510±255 cal. yr BP (OZM369) (Table 1; see also, Table S1). Three U-Th age determinations were attempted on ∼25 mg sub-samples of the flowstone attached to the LL 1 vault (Table 2). Two of these were too contaminated with detrital Th from cave sediments to allow calculation of useable age estimates, but were able to be used to derive a robust estimate of initial ^230^Th/^232^Th activity in this contaminating phase (0.82±0.20). The remaining less-contaminated age determination has been corrected using this figure to provide an absolute age of 7.8±0.5 ka (UMB03650) for the flowstone (Table 2). The flowstone must have formed after the skeleton was deposited, but its dating confirms the Pleistocene-Holocene transition age based on radiocarbon dating of charcoal.

**Table 1:** Radiocarbon age calculations (arranged by depth: see also, Table S1).†

Lab code	Sample code	Stratigraphic	Conventional	Radiocarbon	Mean Calibrated	Depth
		Unit	Radiocarbon Age	Age Error	Age	
			(Years BP)	(±1σ, years)	(years BP, ±2σ error)	(m)
Longlin						
ANSTO	OZM369	-	10014	64	11510±255	-
Maludong						
ANSTO	OZM870	BRRS	11425	50	13290±125	−0.192
ANSTO	OZM143	RS	11527	51	13380±125	−0.737
ANSTO	OZM154	RS	11675	52	13540±165	−0.894
ANSTO	OZM144	DGCA	11874	49	13720±150	−1.200
ANSTO	OZM145	ORS	11749	49	13590±160	−1.660
ANSTO	OZM146	GAS	12037	54	13890±140	−1.995
ANSTO	OZM147	GAS	11982	78	13840±200	−2.001
ANSTO	OZM155	GAS	12020	54	13880±140	−2.020
ANSTO	OZM148	GAS	12137	51	13990±165	−2.348
ANSTO	OZM153	GAS	12430	57	14560±425	−2.398
ANSTO	OZM149	ALROC	12304	59	14310±340	−2.919
ANSTO	OZM150	OROC	13490	65	16630±270	−3.313
ANSTO	OZM151	LGAC	13683	62	16820±185	−3.500
ANSTO	OZM152	BASE	14699	65	17830±240	−3.900

† BP = Before Present (defined as 1950).

**Table 2:** Uranium series results and age calculations.¶

Sample	Lab No. &	U (ngg^−1^)	[^230^Th/^238^U]	[^234^U/^238^U]*	[^232^Th/^238^U]	[^230^Th/^232^Th]	Age	[^234^U/^238^U]_i_ ‡
	Date						(ka)†	
Longlin-1	UMB03649	165	0.775(09)	1.471(05)	1.0317(281)	0.8	−15(42)	1.453(51)
	Oct-2010							
Longlin-2	UMB03650	94	0.146(04)	1.758(08)	0.0321(004)	4.5	7.8(0.5)	1.775(08)
	Oct-2010							
Longlin-3	UMB03651	236	1.446(11)	1.241(05)	1.5956(180)	0.9	-	-
	Oct-2010							

¶ Numbers in brackets are 95% uncertainties of the given least significant figures.

* Activity ratios determined after Hellstrom [53] using the decay constants of Cheng et al. [54].

† Age in kyr before present corrected for intial ^230^Th using eqn. 1 of Hellstrom [55] with [^230^Th/^232^Th]_i_ of 0.82±0.20 for Longlin.

‡ Initial [^234^U/^238^U] calculated using corrected age.

**Figure 2:**
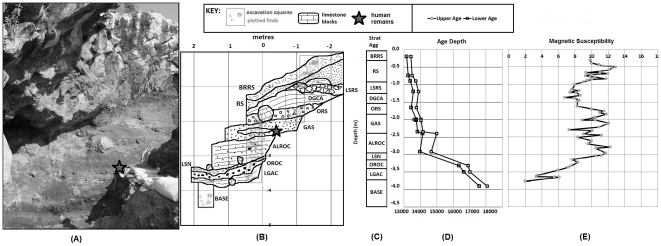
Maludong site. (A) stratigraphic sequence; (B) GIS plotted stratigraphy based on total station data and indicating excavation units and plotted finds; (C) stratigraphic aggregates; (D) Upper and Lower age limit of calibrated radiocarbon ages; and (E) magnetic susceptibility record (×10^−6^ m^3^ kg^−1^).

During the original excavation at Maludong three stratigraphic layers were identified [19]. However, in our recent research on the remaining ∼3.7 m section at the site we identified 11 distinct stratigraphic aggregates (Figure 2). AMS radiocarbon ages from 14 charcoal samples were used to determine an age versus depth profile (Table 1, Figure 2; see also, Table S1), providing unambiguous absolute ages for the stratigraphic units recognised at Maludong. Radiocarbon dating of bone was unsuccessful due to a lack of preserved collagen. A magnetic susceptibility record corroborates the stratigraphically coherent and internally consistent radiocarbon-based chronology for the site, indicating that the dated charcoal was deposited at the same time as its enveloping sediments (Figure 2; see also, Text S1).

Calibrated radiocarbon ages show that the entire sequence spans the interval 17,830±240 cal. yr BP (OZM152) to 13,290±125 cal. yr BP (OZM870) (Table 1). All of the human remains were recovered from within a series of deposits dating from 14,310±340 cal. yr BP (OZM149; 292 cm depth) to 13,590±160 cal. yr BP (OZM145; 166 cm depth), a period of about 720 years. Moreover, the high fine-grained ferrimagnetic content of the deposits (Text S1), with their high magnetic susceptibility, suggests these were formed under warm, wet conditions, consistent with the Bølling-Allerød interstadial (∼14.7-12.6 ka [20]). Human remains recovered *in situ* during the 2008 excavation and a reasonably complete calotte (specimen MLDG 1704) derived from a subsection of these deposits dated between 13,990±165 cal. yr BP (OZM148; 235 cm) and 13,890±140 cal. yr BP (OZM146; 200 cm) (Figure 2).

### Morphological description and comparison

A full list of human remains recovered from Longlin and Maludong is provided in Table 3. Here we describe and compare the cranial, mandibular and dental remains, as they are the most informative with respect to evolution and systematics. Details of comparative samples are provided in Table 4 (see also data sources, Text S2). The grade term ‘modern’ is used interchangeably with *H. sapiens sensu stricto* (i.e. beginning with Omo-Kibish 1, Herto and other so-called anatomically modern humans to recent humans), while ‘archaic’ grade refers to all other hominin specimens/taxa.

**Table 3:** Human remains recovered from Longlin and Maludong.

Locality	Catalogue No.	Description
Longlin	LL	
	1	Partial cranium with left I^1^-M^1^; right I^2^, fragment of M^1^, partial mandible with left I_1_, canine, P_3_, M_2_-M_3_; right I_1_-I_2_, canine, P_3_-P_4_ and M_2_, several isolated tooth fragments, almost complete left half of axis (vertebra C2), proximal ulna fragment and several rib fragments
Maludong	MLDG	
	1678	Femur, proximal ∼⅓^rd^
	1679	Mandible, right body fragment and ramus, M_2_ and M_3_ crowns
	1704	Cranium, calotte
	1705	Cranial vault fragments
	1706	Mandible, right hemi-mandible, no dentition
	1707	Manual phalanx
	1708	Cranium, zygomatic fragment
	1710	Ulna, proximal fragment
	1711	Manual phalanx, proximal fragment
	1712	Rib fragment
	1713	Cranium, maxillary body fragment, no dentition
	1714	Rib fragment
	1715	Rib fragment
	1716	Rib fragment
	1717	Femur, ∼½ head
	1718	Sacrum, partial
	1722–1730	Parietal fragments
	1731–1733	Frontal fragments
	1734	Occipital fragment
	1735–1739	Parietal fragments
	1740	Occipital fragment
	1741	Parietal fragment
	1742–1744	Parietal fragments
	1745	Occipital fragment
	1746	Frontal fragment
	1747	Left M^3^
	1748	Left upper partial premolar (P^3^)
	1749–1750	Sternum, ∼½ of manubrium and ∼½ of body
	1751	Left partial I^2^
	1756	Sacrum, partial

**Table 4:** Cranial series employed in comparisons (where data were compiled by the present authors: see Text S2 for a list of data sources for metrical and morphological data and dating estimates).

Sample Taxon &	Sample	Site/series	Estimated
Region	Abbrev.		Age (kya)
Early *Homo sapiens*			
East Asian	EAEHS	Baojiyan, Chuandong, Dhongzhongyan, Du'an, Gua Gunung, Hang Cho, Huanglong PA 842, Liujiang, Mai Da Nuoc, Minatogawa 1, 4, Moh Khiew, Tubo, Nalai, Upper Cave Zhoukoudian 101, 103, Wushan, Wadjak	∼67-10
European	EUEHS	Barma Grande unnumbered, 1, 2, Combe Capelle, Cotte de St. Brelade, Cro Magnon 1, 2, 3, Mladec 1, 2, 5, 6, Predmost 1, 3, 4, 9, 10, 14, Grotte de Enfants 4, 5, 6	∼32->20
West Asian	WAEHS	Skhul 2, 4, 5, 6, 9, Qafzeh 1, 2, 3, 5, 6, 7, 9	∼173-36
African	AFEHS	Herto BOU-VP-16/1 and Omo-Kibish 1	∼195-150
*Homo neanderthalensis*	NEAND	Amud 1, Arcy-sur-Cure, Ehringsdorf 1, 2, Forbe's Quarry, Gibraltar 1, Guattari 1, Krapina C, D, E, 3, 4, 6, 16, 27/28, 34.1, Krapina Par. 5, Par. 20, Par. 21, Par. 32, Kulna, La Chapelle, La Ferrassie 1, Le Moustier, La Quina 5, 13, Le Moustier, Monte Circeo, Neandertal, Saccopastore 1, Sal'a 1, Shanidar 1, 2, 4, 5, Spy 1–3, Tabun 1, Vindija VI 204, 261, 293, 284–230–255–256, VI 224, VI 227, VI 261	>250-∼32
East Asian Middle Pleistocene			
archaic Hominins	EAMPH	Dali, Jinniushan, Maba, Xujiayao	∼195->104
*Homo erectus sensu stricto*	ERECT	Buku, Chenjiawo, Gongwangling, Hexian, Jianshi PA504, PA502 and PA503, Luonan, Nanjing, Ngandong 1, 3, 5, 6, 7, 9, 10, 11, 12, 14, Sangiran 1b, 2, 3, 4, 9, 10, 12, 17, 18, 21, 22, 38, Sb 8103, T2, Sumbangmacan 1, 3, 4, Trinil II, Wushan, Yiyuan, Yunxian, Xichuan, Zhoukoudian 1, 2, 3, 4, 5, 6, 10–13, 18, 20–33, 43–52, 69, 70–74, 80–95, 106–117, 131, 134, 136, 138, 140, 143–144, 146, Zhoukoudian reconstruction	<1600-143
Howells samples			
East Asian		Ainu, Andaman, Atayal, Buriat, Guam, Hainan, N Japan, Philippines, S Japan	Recent
Australian		Australia, Tasmania	Recent
European		Berg, Norse, Zalavár	Recent
African		Bushman, Dogon, Teita, Zulu	Recent

#### Preservation

**Figure 3:**
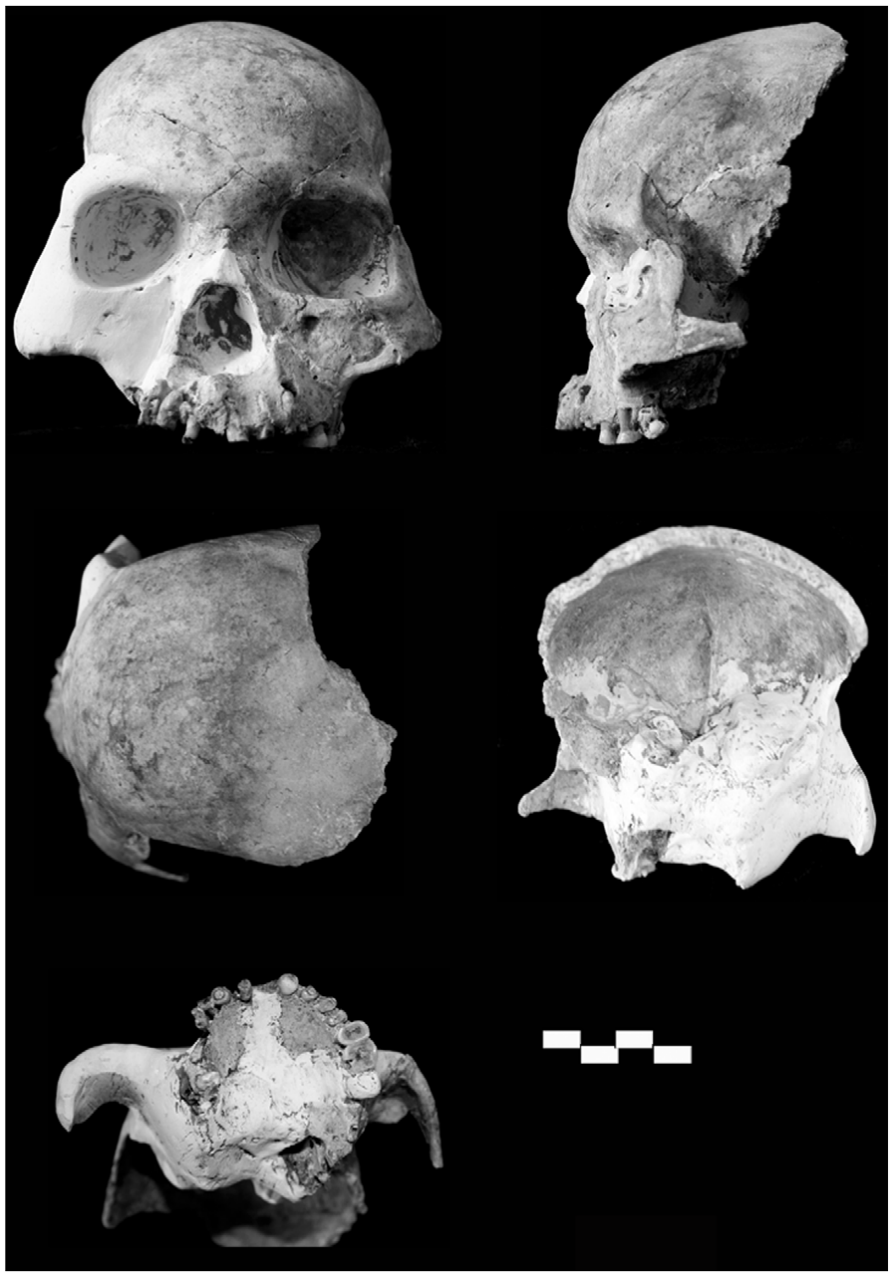
Longlin 1 partial skull (each bar = 1 cm).

**Figure 4:**
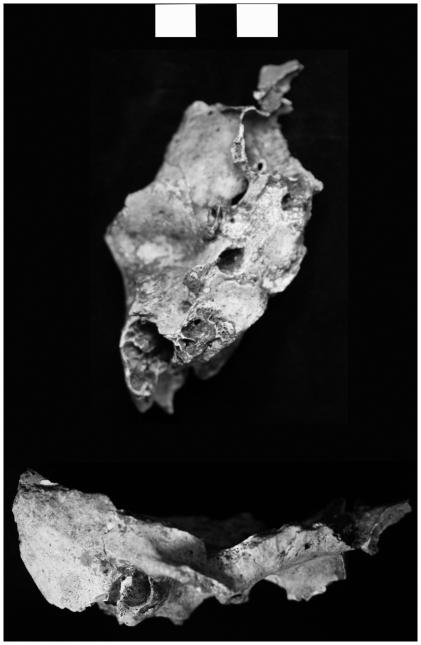
Longlin 1 temporal fragment (each bar = 1 cm).

The LL 1 partial skull (Figure 3) preserves a mostly complete frontal squama with left supraorbital margin, but lacks the right lateral supraorbital part and zygomatic process. The superior section of the nasal bones and superomedial orbital walls are present, as are the left and right frontal processes of the maxillae. Most of the left facial skeleton is present and comprises a nearly complete zygomatic process, alveolar process from mid-line to M^1^, and a largely intact left zygomatic. The right maxilla is incomplete save much of the lateral margin of the piriform aperture and alveolar process. What remains has been rotated ∼45° from the median sagittal plane owing to post-burial compression. The left side (MSP to lateral) is largely free of distortion, with the landmarks *prosthion* and *nasospinale* readily identifiable. The tip of the anterior nasal spine is broken away, but its base is easily discerned. The bony palate lacks most of the left and right palatine processes. The morphology of the preserved left maxillary tuberosity is consistent with M^3^ agenesis. Parts of the sphenoids, anterior occipital, including anterior margin of the foramen magnum, partial left occipital condyle and basioccipital clivus remain. The temporal fragment (Figure 4) preserves a section of the squama, the base of the mastoid process (tip broken away), tympanic part with a damaged external acoustic meatus, mostly complete and pathologically unmodified mandibular fossa, base of the styloid process, vaginal process and stylomastoid foramen, large carotid canal, preserved foramen lacerum, foramen ovale and foramen spinosum, and a largely intact petrous part.

**Figure 5:**
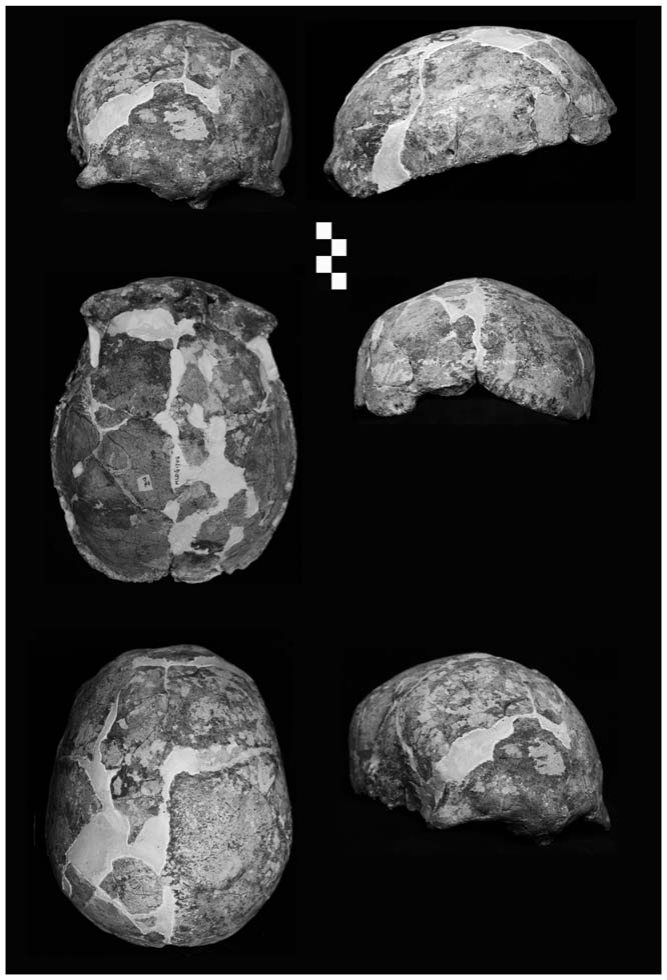
Maludong 1704 calotte (each bar = 1 cm).

The MLDG 1704 calotte (Figure 5) comprises mostly complete frontal and paired parietal bones, but lacks its occipital, temporals and most of the sphenoids, as well as the entire viscerocranium. Evidently the specimen lost its base and facial skeleton owing to anthropogenic alteration, with cut-marks seen along the walls of the vault and on the zygomatic process. Its preserved morphology is unaffected by this alteration. The specimen is free from post-deposition distortion as indicated by visual inspection and scrutiny of CT-scans.

**Figure 6:**
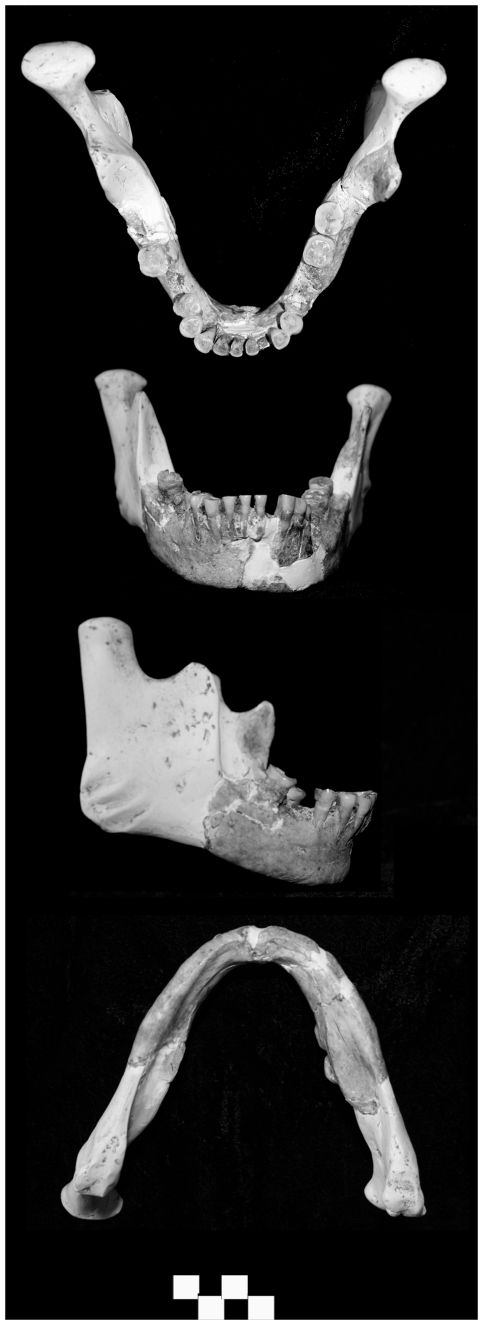
Longlin 1 mandible (scale bar = 1 cm).

The LL 1 mandible (Figure 6) and two partial mandibles recovered from Maludong (MLDG 1679 and MLDG 1706: Figure 7) are also compared. Specimen LL 1 comprises a largely complete body, but is missing its left ramus save the root and coronoid process, and lacks the entire right ramus. The position of the take-off of the left ramus relative to M_3_ makes clear that a retromolar space would have been present (M_3_ being uncovered [21]). The external surface of the symphysis has been displaced superiorly such that the bone is out of alignment with the alveolar process. This makes accurate assessment of chin development problematic. The left alveolar part retains the roots and crowns of I_1_, canine, P_3_, partial P_4_, M_2_ and partial M_3_. The first molar is missing and the alveolar bone shows signs of ante-mortem tooth loss with resorption and new bone growth/remodelling. Much of the right body is preserved and retains the mental foramen, I_1_-P_4_ and M_2_ roots and crowns. The right M_1_ seems again to have been lost ante-mortem, with signs of remodelling of the alveolar bone. The transverse tori are somewhat thickened such that the internal surface of the symphysis is not vertical, a small internal plane being present.

**Figure 7:**
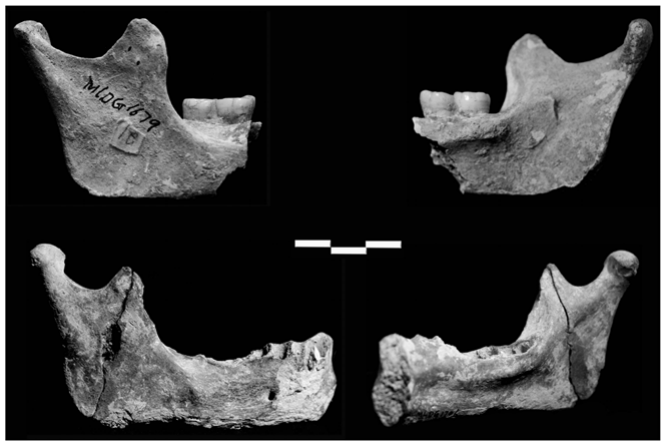
Maludong mandibles MLDG 1679 (left) and MLDG 1706 (right) (scale bar = 1 cm). NB: MLDG 1706 is broken through its symphysis just lateral to the MSP.

Specimen MLDG 1679 (Figure 7) comprises a right mandibular body fragment preserved from just anterior to M_2_, with intact M_2_-M_3_ crowns and roots, and including a complete ramus in two pieces. The internal morphology of the body and ramus is well preserved including the mandibular foramen, pterygoid surface, and coronoid and condylar parts (the former having been modified somewhat by osteoarthritis).

Specimen MLDG 1706 (Figure 7) is a right hemi-mandible, broken just lateral to the symphysis (left side), through to a mostly complete ramus. The body is damaged (abraded) along its inferior border, while scoring marks the external surface of the symphysis. No dental crowns were recovered with the specimen, but all of the alveoli are open and lack signs of bony remodelling, indicating a full (adult) set of dentition would probably have been present about the time of death. Externally, the mental foramen is present and well preserved. Internally, the morphology of the surface of the body and ramus is clear: the symphysis expresses enlarged tori, the mandibular foramen is clear, and the pterygoid surface and coronoid and condylar parts are well preserved (the former being modified slightly by osteoarthritis).

#### Supraorbital region

The supraorbital part of LL 1 is conspicuous, with a well-developed glabella. It lacks the obvious signs of division typically seen among modern humans (i.e. lacks a dividing sulcus between medial and lateral parts), but it does thin in the vertical dimension mediolaterally. The supraorbital torus of MLDG 1704 is marked by a strongly developed glabella and superciliary ridges, but also thins laterally. Its supraorbital part is, however, divided into medial and lateral components by a distinct sulcus, being bipartite in form. The presence of a supraorbital torus is a condition rarely seen in recent humans [22], but is more frequent among Pleistocene *H. sapiens* [23]. A bipartite supraorbital like that seen in MLDG 1704 is characteristic of *H. sapiens* and distinguishes it from archaic taxa [23].

Table 5 compares supraorbital projection and vertical thickness dimensions. Supraorbital projection at the medial location [24] is moderate in LL 1 (11 mm), but high in MLDG 1704 (17 mm, measured in MLDG 1704 on CT-scans). Longlin resembles European early *H. sapiens* (or EUEHS) in this regard (13±3 mm; *z*-score adjusted for the size of the comparison sample [25] −0.63), while MLDG 1704 is identical to West Asian early *H. sapiens* (or WAEHS) crania (i.e. Skhul and Qafzeh: mean 17 mm). Values for both specimens are below the *H. neanderthalensis* (or NEAND) mean (20±2 mm), the difference from LL 1 being significant (*z*-score −4.27, *p*0.001; MLDG 1704 *z*-1.42). At mid-orbit [24], supraorbital projection is comparatively weak in LL 1 (11 mm; EUEHS *z*-1.58), but moderate in MLDG 1704 (16 mm), and identical to the EUEHS mean (Table 5). In contrast, mid-orbit projection [24] is strong in WAEHS (20 mm) and NEAND (22±2 mm; MLDG 1704 *z*-2.97, *p*0.002; LL 1 *z*-5.44, *p*<0.0001). At the lateral position [24], projection in LL 1 is similar to EUEHS (20±3 mm; *z*-0.32), while in MLDG 1704 it is strong (∼23 mm; EUEHS *z*0.95), the specimen closely resembling WAEHS (24 mm, n4) and NEAND (24±2 mm; *z*-0.49; LL 1 *z*-2.47, *p*0.009).

**Table 5:** Bone thickness measurements compared (significant *z*-scores in bold).¶

	LL	MLDG	EUEHS	WAEHS	NEAND	EAMPH	ERECT
	1	1704					
Supraorbital projection							
1. Medial	11.0	17.0	13±3(9)	17(4)	20±2(9)	-	-
			(8–18)	(11–21)	(18–23)		
*z-*score/*p* LL 1			−0.63/0.27	-	**−4.27/0.001**	-	-
*z-*score/*p* MLDG 1704			1.26/0.13	-	−1.42/0.09	-	-
2. Midorbit	11.0	16.0	16±3(9)	20(4)	22±2(43)	-	-
			(8–19)	(16–25)	(17–25)		
*z-*score/*p* LL 1			−1.58/0.07	-	**−5.44/<0.00001**	-	-
*z-*score/*p* MLDG 1704			n.a.	-	**−2.97/0.002**	-	-
3. Lateral	19.0	23.0	20±3(9)	24(4)	24±2(35)	-	-
			(15–23)	(21–26)	(21–25)		
*z*-score/*p* LL 1			−0.32/0.37	-	**−2.47/0.009**	-	-
*z*-score*/p* MLDG 1704			0.95/0.18	-	−0.49/0.31	-	-
Supraorbital vertical thickness						-	-
4. Medial	15.0	16.5/17.0*	17±3(11)	18±3(7)	17±3(9)	-	-
			(12–24)	(14–22)	(15–22)		
*z*-score/*p* LL 1			−0.64/0.26	−0.94/0.19	−0.63/0.27	-	-
*z*-score/*p* MLDG 1704			−0.08/0.46	−0.39/0.35	−0.08/0.46	-	-
5. Midorbit	7.0	10.0/13.0*	5±2(11)	8±3(7)	10±2(48)	-	-
			(6–10)	(5–11)	(8–12)		
*z*-score/*p* LL 1			0.96/0.18	−0.31/0.38	−1.48/0.07	-	-
*z*-score*/p* MLDG 1704			**3.11/0.005**	1.09/0.15	0.74/0.23	-	-
6. Lateral	7.0	7.0/6.5*	8±1(11)	10±3(7)	12±2(42)	-	-
			(6–10)	(6–14)	(10–14)		
*z*-score/*p* LL 1			−0.96/0.18	−0.94/0.19	**−2.47/0.008**	-	-
*z*-score/*p* MLDG 1704			−1.20/0.12	−1.01/0.17	**−2.59/0.006**	-	-
Vault thickness							
7. Bregma	10.0	7.0	7±3(8)	7(2)	7±1(13)	8(4)	9±2(22)
			(4–12)	(6–8)	(4–9)	(7–9)	(6–16)
*z*-score/*p* LL 1			0.94/0.18	-	**2.89/0.006**	-	0.49/0.31
*z*-score/*p* MLDG 1704			n.a.	-	n.a.	-	−0.98/0.16
8. Parietal eminence	-	7.6/6.4*	6±1(16)	8±2(9)	8±2(30)	10±2(5)	10±2(16)
			(4–10)	(5–11)	(5–17)	(7–13)	(5–16)
*z*-score/*p* LL 1			-	-	-	-	-
*z*-score/*p* MLDG 1704			0.97/0.17	−0.47/0.32	−0.47/0.32	−1.37/0.12	−1.46/0.08

¶ Above the line *μ±σ*(*n*), below the line (*min.-max.*); *z*-tests corrected for small comparative sample size; Bonferroni correction not employed as per [56]; sample abbreviations and compositions see Table 4; data sources see Text S2.

* Mean of left and right used in *z*-test.

Vertically thickness of the supraorbital at the medial location [24] is similar in LL 1 (15 mm) and MLDG 1704 (L 16.5/R 17 mm) to EUEHS (17±3 mm; LL 1 *z*0.64; MLDG 1704 *z*-0.08) and NEAND (17±3 mm; LL 1 *z*-0.63; MLDG 1704 *z*-0.08). Their values are, however, slightly reduced compared to WAEHS (18±3 mm; *z*-0.94, *z*-0.39). Mid-orbit thickness [24] is moderate in LL 1 (7 mm) and similar to WAEHS (8±3 mm; *z*-0.31), but well below the NEAND mean (10±2 mm; *z*-1.48). In MLDG 1704, thickness at this location is marked (10/13 mm), and while exceeding mean values for all comparative samples, it is most similar to NEAND (*z*0.74; contrasting with EUEHS *z*3.11, *p*0.005; and WAEHS *z*1.09). Finally, vertical thickness at the lateral location [24] is comparatively thin in LL 1 (7 mm) and MLDG 1704 (7/6.5 mm). They both closely resemble the laterally thin supraorbitals of EUEHS (8±1 mm; LL 1 *z*-0.96; MLDG 1704 *z*-1.20) and WAEHS (10±3; LL 1 *z*-0.94; MLDG 1704 *z*-1.01). In contrast, NEAND tori are laterally thick (12±2 mm; LL 1 *z*-2.47, *p*0.008; MLDG 1704 *z*-2.59, *p*0.006).

#### Vault thickness

Vault thickness measurements are presented for LL 1 and MLDG 1704 and compared in Table 5. At *bregma*, LL 1 has a thick vault (10 mm), being most similar to the *H. erectus* (ERECT) mean (9±2 mm; *z*0.49). While its value is within one standard deviation unit of the EUEHS sample mean (7±3 mm; *z*0.94), it is significantly different to the NEAND mean (7±1 mm; *z*2.89, *p*0.006). Thickness at *bregma* in MLDG 1704 (7 mm) is identical to mean values for EUEHS, WAEHS and NEAND (Table 5). At the parietal eminence, thickness in MLDG 1704 (7.6/6.4 mm) is within one standard deviation unit of means for EUEHS (6±1 mm, *z*0.97) and WAEHS (both 8±2 mm, *z*-0.47), but distinct from the means of East Asian Middle Pleistocene (archaic) hominins (or EAMPH) and ERECT (both 10±2 mm; *z*-1.37 and *z*-1.47).

#### Vault dimensions

Tables 6–7 compare a range of vault measurements for LL 1 and MLDG 1704 with various Pleistocene modern (Table 6) and archaic hominin (Table 7) samples. A comparison of African early *H. sapiens* (or AFEHS) and NEAND helps to sort the polarities of features found in Eurasian samples. The Herto (BOU-VP-16/1) cranium possesses a large endocranial volume (ECV) (1450 cm^3^). It is, however, only slightly enlarged compared with NEAND (1407±172 cm^3^). In contrast, the greatly enlarged ECV of WAEHS (Skhul-Qafzeh: 1556±25 cm^3^) is significantly larger than Herto (*z*-3.87, *p*0.008) and NEAND (*t*-5.44, *p*0.002), indicating that ECV enlargement is a derived characteristic of Pleistocene Eurasian *H. sapiens*. Reconstructed ECV for MLDG 1704 (∼1327 cm^3^: Figures S1, S2, S3, S4) is small in comparison with all *H. sapiens* sample means: its value sits outside of (below) the ranges of WAEHS (*z*-8.36, *p*0.0005) and EUEHS (*z*-1.71, *p*0.05). ECV for East Asian early *H. sapiens* (or EAEHS) (1407±146 cm^3^; MLDG *z*-0.51) is identical to the NEAND mean (Table 7). Among all samples, MLDG 1704 is closest to these latter sample means. Thus, MLDG 1704 shares with EAEHS and NEAND a reduced ECV. This reduction contrasts late Pleistocene humans in East Asia with earlier Eurasian and African modern humans with their large ECVs.

**Table 6:** Vault measurements (cm^3^, mm) and indices (%) compared to Pleistocene modern humans (significant *z*-scores in bold).¶

Measurement	LL	MLDG	EAEHS	EUEHS	WAEHS	AFEHS§
Abbrev. (Martin No.)	1	1704				
1. Endocranial volume	-	(1327)	1407±146(7)	1566±134(12)	1556±25(5)	1450(1)
ECV			(1170–1567)	(1375–1880)	(1518–1587)	-
*z*-score/*p* MLDG 1704			−0.51/0.31	−1.71/0.05	**−8.36/0.0005**	-
2. Frontal chord	112	116	112±6(7)	116±6(17)	113(4)	127.5(2)
FRC* (M29)			(105–119)	(91–111)	(106–118)	(124–131)
*z*-score/*p* LL 1			n.a.	−0.65/0.26	-	-
*z*-score/*p* MLDG 1704			0.62/0.27	n.a.	-	-
3. Frontal arc	134	133	130±5(8)	135±7(9)	128(4)	153(1)
FAA (M26)			(121–136)	(121–148)	(118–133)	-
*z*-score/*p* LL 1			0.75/0.23	−0.14/0.44	-	-
*z*-score/*p* MLDG 1704			0.57/0.29	−0.27/0.39	-	-
4. Minimum frontal breadth	94	95	99±5(7)	101±5(17)	103±5(6)	112(1)
MFB (M9)			(95–109)	(91–111)	(96–110)	-
*z*-score/*p* MLDG 1704			−0.75/0.24	−1.17/0.13	−1.48/0.09	-
5. Maximum frontal breadth	-	125	120(4)	124±7(17)	119±4(5)	120(1)
XFB* (M10)			(112–129)	(107–139)	(114–125)	-
*z*-score/*p* MLDG 1704			-	0.14/0.44	1.37/0.12	-
6. Parietal chord	-	107	117±4(7)	120±7(19)	121(4)	125(2)
PAC* (M30)			(111–122)	(107–143)	(112–129)	(121–129)
*z*-score/*p* MLDG 1704			**−2.34/0.02**	**−1.81/0.04**	-	-
7. Parietal arc	-	123	132±9(8)	134±9(19)	133(4)	135(2)
PAA (M27)			(120–150)	(117–157)	(120–145)	(130–140)
*z*-score/*p* MLDG 1704			−0.94/0.18	−1.19/0.12	-	-
8. Maximum parietal breadth	-	141	138±6(7)	143±7(19)	145±3(7)	145(1)
MPB (M8)			(131–145)	(131–154)	(140–148)	-
*z*-score/*p* MLDG 1704			0.47/0.32	−0.28/0.39	−1.25/0.12	-
9. Parietal/frontal chord index	-	92	104±5(7)	104±7(17)	107(4)	98(2)
(M30/M29)			(95–112)	(94–117)	(98–113)	(92–104)
*z*-score/*p* MLDG 1704			**−2.24/0.03**	−1.67/0.05	-	-
10. Parietal/frontal arc index	-	92	102±8(8)	99±7(19)	107(3)	85(1)
(M27/M26)			(94–115)	(87–110)	(102–111)	-
*z*-score/*p* MLDG 1704			−1.18/0.13	−0.97/0.17	-	-
11. Frontal constriction index	-	76	83(4)	82±2(16)	86±3(5)	93(1)
(M9/M10)			(76–89)	(79–86)	(80–88)	-
*z*-score/*p* MLDG 1704			-	**−2.91/0.005**	**−3.04/0.01**	-

¶ Fossil values in round brackets are estimates; above the line *μ±σ*(*n*), below the line (*min.-max.*); *z*-tests corrected for small comparative sample size; Bonferroni correction not employed as per [56]; sample abbreviations and compositions see Table 4; data sources see Text S2.

§ Mostly comprises measurements of Herto BOU-VP-16/1.

* Equivalent to measurements of Howells [57] and employing his abbreviation.

**Table 7:** Vault measurements (cm^3^, mm) and indices (%) compared to archaic hominins (significant *z*-scores in bold).¶

Measurement	LL	MLDG	NEAND	EAMPH	ERECT
Abbrev. (Martin No.)	1	1704			
1. Endocranial volume	-	(1327)	1407±172‡(28)	1255(2)	1028±127$ ^,^ ¥(16)
ECV			(1172–1740)	(1120–1390)	(815–1251)
*z*-score/*p* MLDG 1704			−0.46/0.32	-	**2.28/0.01**
2. Frontal chord	112	116	112±6†(18)	116(1)	112±6£(12)
FRC* (M29)			(98–123)	-	(99–120)
*z*-score/*p* LL 1			n.a.	-	n.a.
*z*-score/*p* MLDG 1704			0.65/0.26	-	0.64/0.26
3. Frontal arc	134	133	123±9‡(12)	134(1)	125±7$(12)
FAA (M26)			(107–135)	-	(110–135)
*z*-score/*p* LL 1			1.17/0.13	-	1.24/0.12
*z*-score/*p* MLDG 1704			1.07/0.15	-	1.10/0.14
4. Minimum frontal breadth	94	95	103±5†(21)	114(1)	93±10$ ^,^ ¥(23)
MFB (M9)			(97–112)	-	(73–109)
*z*-score/*p* MLDG 1704			−1.56/0.06	-	0.20/0.42
5. Maximum frontal breadth	-	125	122±6†(13)	-	118±8$ ^,^ ¥(23)
XFB* (M10)			(108–128)	-	(102–123)
*z*-score/*p* MLDG 1704			0.48/0.33	-	0.86/0.20
6. Parietal chord	-	107	108±4‡(13)	110(2)	100±5$ ^,^ ¥(21)
PAC* (M30)			(102–114)	(107–113)	(86–108)
*z*-score/*p* MLDG 1704			−0.24/0.40	-	1.37/0.09
7. Parietal arc	-	123	115±5‡(10)	117.5(2)	106±5$ ^,^ ¥(22)
PAA (M27)			(110–126)	(114–121)	(92–113)
*z*-score/*p* MLDG 1704			1.53/0.08	-	**3.33/0.001**
8. Maximum parietal breadth	-	141	148±7†(16)	148.5(2)	142±6¥(21)
MPB (M8)			(138–157)	(148–149)	(130–153)
*z*-score/*p* MLDG 1704			−0.97/0.17	-	−0.16/0.43
9. Parietal/frontal chord index	-	92	98±8†(10)	92(1)	89±7(11)
(M30/M29)			(89–116)	-	(81–104)
*z*-score/*p* MLDG 1704			−0.72/0.24	-	0.41/0.34
10. Parietal/frontal arc index	-	92	96±10(9)	85(1)	84±5(11)
(M27/M26)			(83–115)	-	(71–91)
*z*-score/*p* MLDG 1704			−0.38/0.35	-	1.53/0.07
11. Frontal constriction index	-	76	86±5(13)	-	82±5(21)
(M9/M10)			(78–94)	-	(72–89)
*z*-score/*p* MLDG 1704			**−1.93/0.03**	-	−1.17/0.12

¶ Fossil values in round brackets are estimates; above the line *μ±σ*(*n*), below the line (*min.-max.*); *z*-tests corrected for small comparative sample size; Bonferroni correction not employed as per [56]; sample abbreviations and compositions see Table 4; data sources see Text S2.

* Equivalent to measurements of Howells [57] and employing his abbreviation.

† *T*-test EUEHS and NEAND mean difference: one-tailed *p*<0.05-0.01.

‡ *T*-test EUEHS and NEAND mean difference: one-tailed *p*<0.001.

£ *T*-test EUEHS and ERECT mean difference: one-tailed *p*<0.05-0.01.

$ *T*-test EUEHS and ERECT mean difference: one-tailed *p*<0.001.

¥ *T*-test NEAND and ERECT mean difference: one-tailed *p*<0.001.

The frontal bone of the earliest modern humans is long (AFEHS frontal chord 124-131 mm, arc 153 mm) and contrasts with the short frontals of NEAND (chord 112±6 mm, arc 123±9 mm). The frontal bones of LL 1 (chord 112 mm, arc 134 mm) and MLDG 1704 (chord 116 mm, arc 133 mm) are moderate in length, being similar to EUEHS and ERECT, but distinguishable from the very long frontals of AFEHS. Minimum frontal breadth is wide in AFEHS (i.e. Herto 112 mm), contrasting with the narrower post-orbitals of NEAND (103±5 mm). This region is even narrower in LL 1 (94 mm) and MLDG 1704 (95 mm), contrasting with the moderately broad anterior frontals of EAEHS (99±5 mm), EUEHS (101±5 mm; *z*-1.36, *z*-1.17), and WAEHS and NEAND (both 103±5 mm; WAEHS *z*-1.67, *z*-1.48; NEAND *z*-1.76, *p*0.04, *z*-1.56, *p*0.06). The LL 1 and MLDG 1704 values are most similar to the mean for ERECT (93±10 mm; *z*0.10, *z*0.20). Maximum frontal breadth, measured at the coronal suture, is moderate in AFEHS and EAEHS (120 mm), and WAEHS (119±4 mm) and NEAND (122±6 mm). MLDG 1704 possesses a broad maximum frontal width (125 mm), in common with EUEHS (124±7 mm, *z*0.14).

The parietal bones of AFEHS are long (chord 125 mm, arc 135 mm) and contrast strongly with the short parietals of NEAND (chord 108±4 mm, arc 115±5 mm). The parietals of MLDG 1704 are short (chord 107 mm, arc 123 mm) by Pleistocene *H. sapiens* standards (Table 6). Its parietal chord is significantly shorter than the EAEHS (117±4 mm; *z*-2.34, *p*0.02) and EUEHS (120±7 mm; *z*-1.81, *p*0.04), and most closely resembles NEAND (*z*-0.24). Its arc dimension is, however, closer to *H. sapiens* (means 132–135 mm; MLDG 1704 z-0.94 to z-1.19) than to archaic species (means 106–117.5 mm; MLDG 1704 *z*1.17 to *z*3.33; with *p*0.001 for ERECT). Broad parietals are characteristic of NEAND (148±7 mm), distinguishing them from *H. sapiens* (means 138–145 mm) and ERECT (142±6 mm). The absolutely narrow bi-parietal breadth of MLDG 1704 (141 mm) is most similar to the mean of EUEHS (143±7 mm, *z*-0.28).

The ratio of parietal/frontal chord and parietal/frontal arc distinguishes samples of Eurasian *H. sapiens* (means: chord 104–107%, arc 99–107%) from NEAND (chord 98±8%, arc 96±10%). The shortened parietals of AFEHS (chord 98%, arc 85%) are, in contrast, a putative ancestral trait shared with NEAND. Thus, parietal sagittal expansion is characteristic of Pleistocene Eurasian *H. sapiens*. For these indices, MLDG 1704 (both 92%) is highly distinct from *H. sapiens* (EAEHS *z*2.24, *p*0.03; EUHS *z*1.67, *p*0.05), and most closely resembles archaic hominins such as East Asian Middle Pleistocene hominins (or EAMPH: chord index 92%), ERECT (chord: 87±7%, *z*0.41) and NEAND (arc: 96±10%, *z*-0.38).

A commonly deployed index of postorbital width is the frontal constriction index, or ratio of minimum/maximum frontal breadth. Its value for MLDG 1704 (76%) is unusually low, and while it sits (just) within the lower part of the range of EAEHS (76–89%), it is most similar to the mean for ERECT (82±5%, MLDG *z*-1.17). In contrast, MLDG 1704 is distinct from mean values for EUEHS (82±2%; *z*-2.91, *p*0.005), WAEHS (83±3%; *z*-3.04, *p*0.01) and NEAND (86±5%; *z*1.93, *p*0.03).

#### Mandibular fossa

Measurements of the left mandibular fossa of LL 1 (Figure 4) are compared in Table 8. Its mandibular fossa is moderate in length (A-P 17 mm), being similar to the WAEHS (Skhul-Qafzeh: 19 mm), ERECT (20±2 mm; *z*-1.04) and EUEHS (21 mm) means. It is, however, significantly different to the means of recent Africans (11±1 mm; *z*5.97, *p*<0.00001), a sample of Pleistocene early *H. sapiens* (comprising Ngaloba, Jebel Irhoud 1 and 2, Singa 1, Omo-Kibish 2, Skhul 4 and 5 [26]) (11±2 mm; *z*2.81, *p*0.01) and NEAND (11±1 mm; *z*5.74, *p*<0.00001). The mandibular fossa of LL 1 is very broad (M-L c31 mm) and lies outside of the range of all comparative samples, being closest to EAMPH (30 mm). Its breadth is significantly different to the means of recent Africans (20±2 mm; *z*5.47, *p*<0.00001), Pleistocene early *H. sapiens* (composition, see above: 23±2 mm; *z*3.74, *p*0.004) and NEAND (21±3 mm; *z*3.19, *p*0.004). Its mandibular fossa is comparatively deep (S-I 13 mm) like ERECT (12±4 mm; *z*0.23), but shallower than EAMPH (17 mm). In contrast, samples of *H. sapiens* exhibit smaller mean values or much shallower fossae (5–6 mm; *z*6.55–7.96; *p*0.0003-<0.00001). Shallow fossae are also characteristic of NEAND (6±2 mm; *z*3.35, *p*0.003).

**Table 8:** Mandibular fossa dimensions (mm) of Longlin 1 compared (significant *z*-scores in bold).¶

	A-P	M-L	S-I
	Length	Breadth	Depth
LL 1	17	(31)	13
Recent Africans*	11±1(99)	20±2(99)	5±1(99)
	(8–14)	(16–27)	(4–8)
*z*-score/*p*	**5.97/<0.00001**	**5.47/<0.00001**	**7.96/<0.00001**
EUEHS*	21(3)	25(3)	6(3)
	(19–25)	(21–30)	(5–7)
WAEHS*	19(4)	27(3)	7(4)
	(17–22)	(26–29)	(5–8)
Pleistocene early *H. sapiens* * ^,^ ‡	11±2(7)	23±2(7)	6±1(7)
	(8–14)	(21–25)	(5–8)
*z*-score/*p*	**2.81/0.01**	**3.74/0.004**	**6.55/0.0003**
NEAND*	11±1(11)	21±3(11)	6±2(11)
	(9–13)	(16–26)	(4–8)
*z*-score/*p*	**5.74/<0.00001**	**3.19/0.004**	**3.35/0.003**
EAMPH§	27(1)	30(1)	17(1)
ERECT§	20±2(6)	25(4)	12±4(6)
	(17–22)	(23–27)	(5–15)
*z*-score/*p*	−1.04/0.10	-	0.23/0.41

¶ Sample abbreviations see Table 4; LL 1 value in round brackets is an estimate; above the line *μ±σ*(*n*), below the line data (*min.-max.*); Bonferroni correction not employed as per [56].

* Comparative sample descriptive statistics from [26].

‡ Composition, see text.

§ Sample compiled by the authors: compositions see Table 4, data sources see Text S2.

#### Facial skeleton

The facial skeleton of LL 1 is unusual compared with early *H. sapiens* in exhibiting strong alveolar prognathism. The mid-face is flat, both at the nasal root and piriform aperture, and zygomatic process of the maxilla. The specimen lacks a canine fossa, but possesses a deep *sulcus maxillaris*. The left zygomatic arch is laterally flared. The zygomatic bone is strongly angled such that its inferior margin sits well lateral to the superior part. The zygomatic tubercle is small and sits lateral to a vertical line projected from the orbital pillar. The anterior section of the masseter attachment is marked by a broad and deep sulcus, but the zygomatic tubercle is small. The anterior wall of the zygomaticoalveolar root is in an anterior position (above P^4^/M^1^). The lateral orbital margin (pillar) exhibits strong transverse incurvation when viewed in lateral aspect. In most of these features, LL 1 displays the putative plesiomorphic condition for later hominins, being highly distinguishable from the modal condition of *H. sapiens*.

Tables 9–10 compare standard measurements and indices of the facial skeleton for LL 1 and a single measurement for MLDG 1704 with Pleistocene modern human (Table 9) and archaic (Table 10) samples. Data for superior facial breadth are unavailable for AFEHS. However, the narrower upper face of NEAND (118±4 mm) distinguishes them from the very broad upper facial skeletons of WAEHS (Qafzeh-Skhul: 123±7 mm). This later morphology is shared by WAEHS with ERECT (123 mm). Contrasting with both of these conditions are later Eurasian samples of *H. sapiens* with their markedly narrow superior facial skeletons (EAEHS and EUEHS: mean 112 mm). A narrow superior facial breadth is a shared condition of LL 1 (c110 mm) and MLDG 1704 (109 mm), both with each other and with later Pleistocene Eurasians. Their values are, however, highly distinct from WAEHS (LL 1 *z*-1.72, *p*0.07; MLDG 1704 *z*-1.85, *p*0.06) and NEAND (LL 1 *z*-1.96, *p*0.03; MLDG 1704 *z*-2.21, *p*0.01).

**Table 9:** Facial skeleton measurements (mm) and indices (%) compared to Pleistocene modern humans (significant *z*-scores in bold).¶

Measurement	LL	MLDG	EAEHS	EUEHS	WAEHS	AFEHS§
Abbrev. (Martin No.)	1	1704				
1. Superior facial breadth	[110]	109	112±5(5)	112±6(11)	123±7(6)	-
SFB (M43)			(106–119)	(102–124)	(96–110)	-
*z*-score/*p* LL 1			−0.37/0.36	−0.32/0.37	−1.72/0.07	-
*z*-score/*p* MLDG 1704			−0.55/0.30	−0.48/0.32	−1.85/0.06	-
2. Bizygomatic breadth	[144]	-	136±5(6)	140±7(10)	148(4)	142(1)
ZYB* (M45)			(128–143)	(129–156)	(140–160)	-
*z*-score/*p* LL 1			1.48/0.09	0.54/0.29	-	-
3. Postorbital constriction index	(66)	-	73±3(6)	73±4(10)	70(4)	79(1)
(M9/M45)			(70–78)	(66–78)	(66–76)	-
*z*-score/*p* LL 1			**−2.16/0.04**	−1.67/0.06	-	-
4. Bimaxillary breadth	[108]	-	105±6(7)	101±8(8)	97(3)	100(1)
ZMB* (M46)			(95–114)	(85–109)	(90–110)	-
*z*-score/*p* LL 1			0.47/0.32	0.82/0.21		-
5. Upper versus mid-facial breadth	98	-	93±7(5)	88±5(5)	77(3)	-
index (M46/M43)			(84–102)	(80–92)	(69–90)	-
*z*-score/*p* LL 1			0.65/0.27	1.83/0.07	-	-
6. Superior facial height	64	-	67±5(8)	68±5(13)	75±3(5)	79(1)
SFH (M48)			(61–75)	(59–79)	(72–79)	-
*z*-score/*p* LL 1			−0.57/0.29	−0.77/0.22	**−3.35/0.01**	-
7. Facial index	(44)	-	50±2(6)	49±4(10)	51(4)	56(1)
(M48/M45)			(46–52)	(44–58)	(49–53)	-
*z*-score/*p* LL 1			**−2.78/0.01**	**−**1.19/0.13	-	-
8. Orbit breadth	45	-	44±2(8)	43±4(14)	45±2(5)	-
ORB (M51)			(42–48)	(38–48)	(42–47)	-
*z*-score/*p* LL 1			0.47/0.32	0.48/0.31	n.a.	-
9. Orbit height	34	-	31±1(7)	30±3(14)	33±3(5)	34(1)
OBH* (M52)			(29–33)	(26–36)	(29–37)	-
*z*-score/*p* LL 1			**2.81/0.01**	1.29/0.11	0.30/0.38	-
10. Orbital index	76	-	71±5(7)	70±7(14)	74±7(5)	-
(M52/M51)			(67–79)	(59–88)	(65–84)	-
*z*-score/*p* LL 1			0.93/0.19	0.83/0.21	0.26/0.40	-
11. Maximum nasal width	(32)	-	28±2(8)	26±2(13)	30±1(5)	29(1)
NLB* (M54)			(25–32)	(22–30)	(28–32)	-
*z*-score/*p* LL 1			1.89/0.05	**2.89/0.006**	1.83/0.07	-
12. Nasal height	45	-	50±4(8)	51±4(11)	42±2(5)	56(1)
NAH (M55)			(46–58)	(43–59)	(42–47)	-
*z*-score/*p* LL 1			−1.18/0.13	−1.44/0.09	1.37/0.12	-
13. Nasal index	(71)	-	56±4(8)	50±6(11)	56±3(5)	-
(M54/M55)			(50–60)	(44–63)	(53–62)	-
*z*-score/*p* LL 1			**3.54/0.004**	**3.35/0.003**	**4.56/0.005**	-

¶ Fossil values in round brackets are estimates, values in square brackets estimated by measuring to the midline and doubling; above the line *μ±σ*(*n*), below the line (*min.-max.*); *z*-tests corrected for small comparative sample size; Bonferroni correction not employed as per [56]; sample abbreviations and compositions see Table 4; data sources see Text S2.

§ Mostly comprises measurements of Herto BOU-VP-16/1.

* Equivalent to measurements of Howells [57] and employing his abbreviation.

**Table 10:** Facial skeleton measurements (mm) and indices (%) compared to archaic hominins (significant *z*-scores in bold).¶

Measurement	LL	MLDG	NEAND	EAMPH	ERECT
Abbrev. (Martin No.)	1	1704			
1. Superior facial breadth	[110]	109	118±4‡(27)	-	123(2)
SFB (M43)			(107–128)	-	(121–125)
*z*-score/*p* LL 1			**−1.96/0.03**	-	-
*z*-score/*p* MLDG 1704			**−2.21/0.01**	-	-
2. Bizygomatic breadth	[144]	-	145±8(6)	148(1)	-
ZYB* (M45)			(130–153)	-	-
*z*-score/*p* LL 1			−0.12/0.45	-	-
3. Postorbital constriction index	(66)	-	74±2(6)	77(1)	71.5(2)
(M9/M45)			(71–77)	-	(69–74)
*z*-score/*p* LL 1			**−3.70/0.006**	-	-
4. Bimaxillary breadth	[108]	-	112±5‡(13)	-	107(2)
ZMB* (M46)			(104–120)	-	(98–116)
*z*-score/*p* LL 1			−0.77/0.22	-	-
5. Upper versus mid-facial breadth	98	-	95±5(10)	-	87(2)
index (M46/M43)			(85–101)	-	(81–93)
*z*-score/*p* LL 1			0.57/0.98	-	-
6. Superior facial height	64	-	87±5‡(13)	74(1)	80.5(2)
SFH (M48)			(107–128)	-	(77–82)
*z*-score/*p* LL 1			**−4.43/0.0004**	-	-
7. Facial index	(44)	-	59±2(6)	50(1)	53(2)
(M48/M45)			(58–61)	-	(52–55)
*z*-score/*p* LL 1			**−6.94/0.0004**	-	-
8. Orbit breadth	45	-	44±3(12)	47(3)	42(2)
ORB (M51)			(40–49)	(44–52)	(40–44)
*z*-score/*p* LL 1			0.32/0.37	-	-
9. Orbit height	34	-	38±1‡(16)	36(3)	37(2)
OBH* (M52)			(36–41)	(34–39)	(34–40)
*z*-score/*p* LL 1			**−3.88/0.0007**	-	-
10. Orbital index	76	-	87±6(11)	71(2)	88(2)
(M52/M51)			(78–98)	(67–76)	(85–91)
*z*-score/*p* LL 1			−1.76/0.05	-	-
11. Maximum nasal width	(32)	-	32±4‡(16)	31(1)	27(2)
NLB* (M54)			(23–39)	-	(24–30)
*z*-score/*p* LL 1			n.a.	-	-
12. Nasal height	45	-	61±3‡(6)	-	50.5(2)
NAH (M55)			(58–66)	-	(48–53)
*z*-score/*p* LL 1			**−4.94/0.002**	-	-
13. Nasal index	(71)	-	55±3(6)	-	56.5(2)
(M54/M55)			(50–59)	-	(56–57)
*z*-score/*p* LL 1			**4.94/0.002**	-	-

¶ Fossil values in round brackets are estimates, values in square brackets estimated by measuring to the midline and doubling; above the line *μ±σ*(*n*), below the line (*min.-max.*); *z*-tests corrected for small comparative sample size; Bonferroni correction not employed as per [56]; sample abbreviations and compositions see Table 4; data sources see Text S2.

* Equivalent to measurements of Howells [57] and employing his abbreviation.

‡ *T*-test EUEHS and NEAND mean difference: one-tailed *p*<0.001.

The facial skeleton of LL 1 is broad. Bizygomatic breadth is estimated to be wide (c144 mm), strongly distinguishing it from EAEHS, the value for LL 1 being outside of (slightly above) its range. Its bizygomatic most closely resembles NEAND (145±8 mm; *z*-0.12), and is similar also to AFEHS (142 mm). A second index of postorbital constriction is the ratio minimum frontal breadth/bizygomatic breadth, providing a more direct measure of the relative size of the temporal fossa. The value for LL 1 is large (66%) by later hominin standards. While it is equal to the minimum value for EUEHS and WAEHS, its value is distant from their means (EUEHS (73±4%; *z*-1.67, *p*0.06; WAEHS 70%). It also contrasts strongly with EAEHS (73±3%; *z*-2.16, *p*0.04) and NEAND (74±2%; *z*-3.70, *p*0.006). In contrast, bimaxillary breadth in LL 1 (108 mm) is most similar to EAEHS (105±6 mm; *z*0.47). While the index of upper/mid-facial (bimaxillary) breadth is high for LL 1 (98%), it is similar to EAESH (93±5%; *z*0.65) and NEAND (95±5%; *z*0.57).

Superior facial height is greatly reduced in LL 1 (64 mm), a condition distinguishing Eurasian early *H. sapiens* from AFEHS (79 mm) and archaic hominins (means: 80.5–87 mm). The superior facial height of LL 1 is in fact significantly shorter than the mean for WAEHS (73±3 mm; *z*-3.35, *p*0.01). Facial shortening is also seen in archaic EAMPH (74 mm), but not to the great extent characterising late Pleistocene *H. sapiens* (but more so than the AFEHS specimen from Herto). The facial index (superior height/bizygomatic breadth) for LL 1 (44%) shows its bony face to be very short relative to breadth. The value in the specimen is not especially close to any sample mean and is significantly different to EAEHS (50±2%; *z*2.81, *p*0.01) and NEAND (59±2%; *z*-6.94, *p*0.0004).

While the left orbit of LL 1 is broad (45 mm), being identical to the WAEHS mean, this measurement has little discriminating power, as LL 1's value lies within one standard deviation unit of EAEHS (44±2 mm), EUEHS (43±4 mm) and NEAND (44±3 mm). In contrast, orbit height is moderate in LL 1 (34 mm), distinguishing the specimen from the short orbits of EAEHS (31±1 mm; *z*2.81, *p*0.01) and EUEHS (30±3 mm; *z*1.29), and tall orbits of NEAND (38±1 mm; *z*-3.88, *p*0.0007). The value for LL 1 is identical to AFEHS and similar to WAEHS (33±3 mm). This combination of a broad and moderately tall orbit results in a moderate orbital index value (76%), being most similar to WAEMH (74±7%; *z*0.26), but distant from NEAND (87±6; *z*-1.76).

The piriform aperture is broad in LL 1 (maximum nasal width 32 mm), being identical to the NEAND mean (Table 10). It is substantially broader than the means for all *H. sapiens* samples: EAEHS 28±2 mm (*z*1.89), EUEHS 26±2 mm (*z*2.89, *p*0.006) and WAEHS 30±1 mm (*z*1.83). Nasal height is short (45 mm), its value being distant from the means of early *H. sapiens* (means 42–51 mm; *z*1.37–1.44). It is, however, significantly different to the NEAND mean (61±3 mm; *z*-4.94, *p*0.002). The nasal index for LL 1 (71%) is large by later Pleistocene hominin standards, its value being significantly different to all comparative sample means (EAEHS *z*3.54, *p*0.004; EUEHS *z*3.35, *p*0.003; WAEHS *z*4.56, *p*0.005; NEAND *z*4.94, *p*0.002).

#### Multivariate cranial comparisons

**Figure 8:**
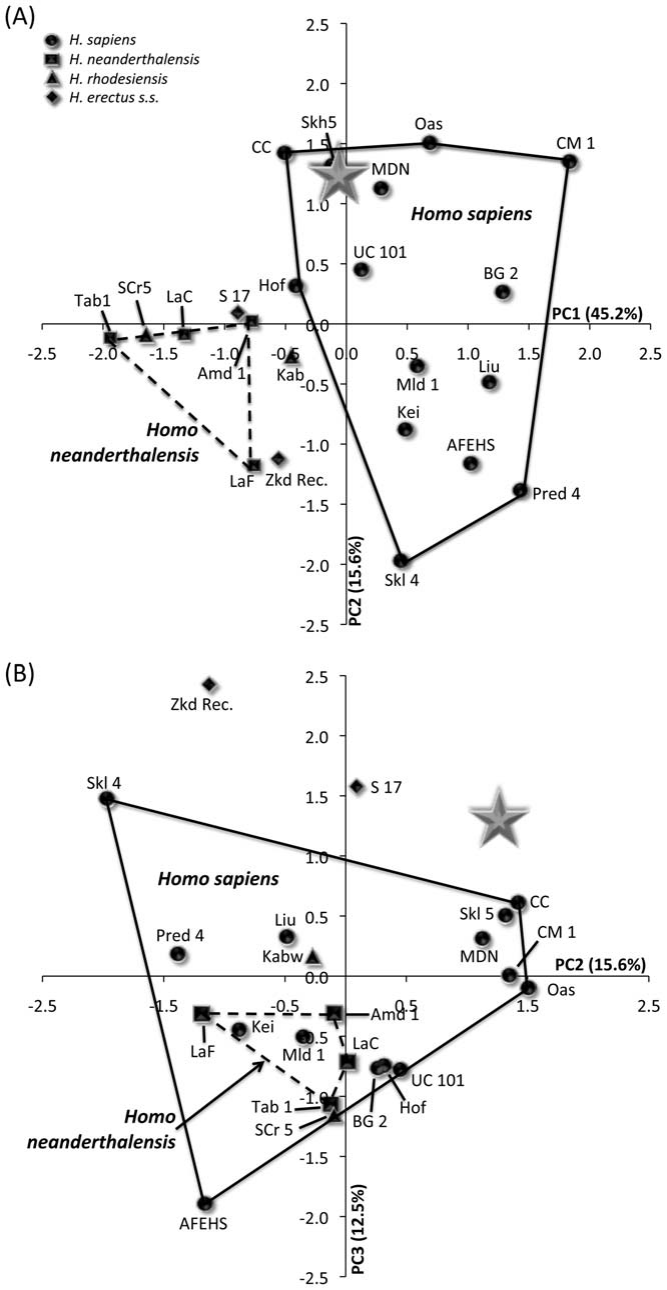
Object plots from principal components analysis including LL 1 and 23 later Pleistocene fossil crania. (A) PC 1 versus 2, and (B) PC 2 versus PC 3 (NB: Gray star = LL 1; AFEHS = African early *H. sapiens*; Amd = Amud; BG = Barma Grande; CC = Combe Capelle; CM = Cro Magnon; Kei = Keilor; Hof = Hofmeyr; Kab = Kabwe; LaC = La Chapelle; LaF = La Ferrassie; Liu = Liujiang; MDN = Mai Da Nuoc; Mld = Mladec; Oas = Oase; Pred = Predmost; S = Sangiran; SCr = Sima de Los Huesos cranium; Skh = Skhul; Tab = Tabun; UC = Upper Cave-Zhoukoudian; and Zkd = Zhoukoudian ERECT).

Table 11 summarises the results of principal components analysis of size-adjusted [27] variables for three sets of analyses comparing LL 1 or MLDG 1704 to fossil specimens. The first analysis included LL 1 (Figure 8) and 22 other crania, employed 9 variables (Table 11), and generated three principal components. For principal component (PC) 1, the highest loading variables were frontal chord and frontal arc, and these were contrasted mostly with measures of facial height (orbit height and upper facial height) and breadth (chiefly nasal breadth) (Table 11). For PC 2, facial breadth (orbit breadth and bimaxillary breadth) accounted for most of the variance (Table 11), while PC 3 contrasted maximum frontal breadth with bizygomatic breadth (Table 11). A bivariate plot of object scores for PC 1 and PC 2 (Figure 8A) shows that PC 1 distinguishes crania belonging to *H. sapiens* from those of NEAND, *H. heidelbergensis sensu stricto*, *H. rhodesiensis* and ERECT. Specimen LL 1 lies within the range of *H. sapiens*, clustering with Skhul 5, Mai Da Nuoc and Combe Capelle. A plot of PC 2 versus PC 3 (Figure 8B) shows the third principal component to distinguish ERECT from all other taxa. In this plot, LL 1 sits in a unique position, well away from all crania owing to a combination of a high positive score for PC 2 and moderate score for PC 3. *Z*-tests of object scores indicate that the difference between LL 1 and the *H. sapiens* mean is not significant for all PCs (PC 1 *z*-0.89; PC 2 *z*0.95; approaching significance for PC 3 *z*1.70, *p*0.05).

**Table 11:** Results of principal components analysis (two highest loading variables for each component in bold).

	Component	Component	Component
	1	2	3
**LL 1**			
*Set 1–9 variables x 23 objects*			
Eigen score	4.07	1.41	1.13
% Variance	45.23	15.63	12.55
Variable loadings			
FRC	**0.956**	0.026	0.083
FAA	**0.949**	0.008	0.040
MFB	0.530	−0.283	**−0.720**
NPH	−0.678	−0.393	−0.125
ORB	0.122	**0.775**	0.094
OBH	−0.725	−0.110	0.272
NLB	−0.801	−0.121	−0.086
ZMB	−0.449	**0.646**	−0.093
ZYB	0.365	−0.355	**0.698**
**MLDG 1704**			
*Set 1–8 variables x 23 objects*			
Eigen score	3.61	2.44	1.11
% Variance	45.09	30.50	13.86
Variable loadings			
FRC	−0.072	**0.953**	−0.147
PAC	**−0.906**	−0.368	−0.136
FAR	−0.235	**0.938**	0.024
PAR	**−0.931**	−0.334	−0.065
MFB	0.582	−0.573	**0.331**
BPB	0.902	0.118	−0.007
XFB	−0.133	0.215	**0.929**
SFB	0.831	−0.120	−0.303
*Set 2–6 variables x 43 objects*			
Eigen score	2.77	1.84	-
% Variance	46.21	30.68	-
Variable loadings			
FRC	0.694	0.581	-
PAC	**−0.890**	0.019	-
FAA	0.496	**0.825**	-
PAA	**−0.878**	0.258	-
MFB	0.494	**−0.782**	-
XFB	0.486	−0.378	-

**Figure 9:**
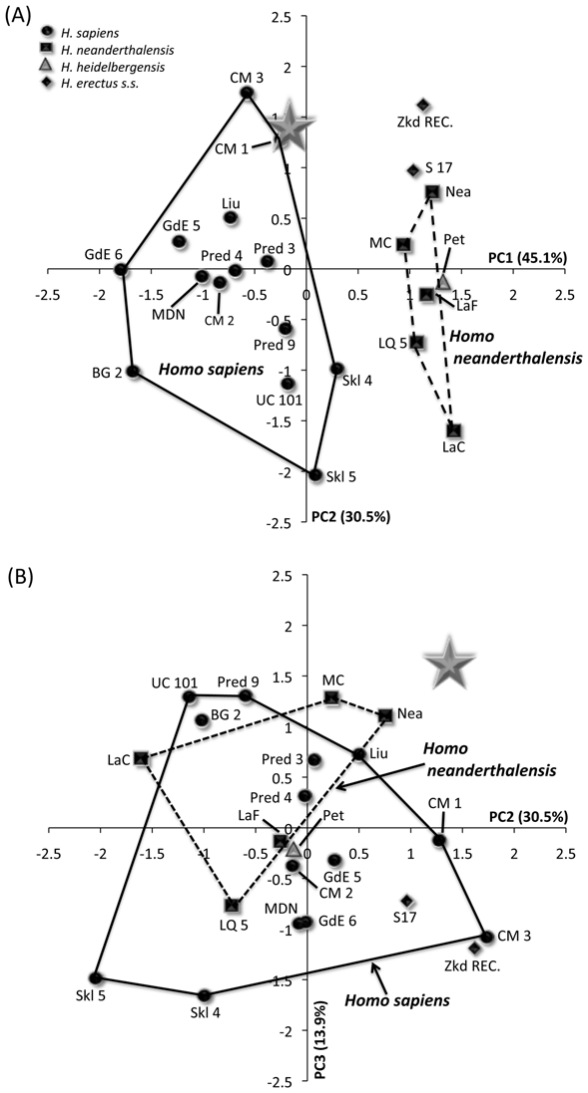
Object plots from principal components analysis including MLDG 1704 and 23 later Pleistocene fossil crania. (A) PC 1 versus 2, and (B) PC 2 versus PC 3 (NB: Gray star = MLDG 1704; BG = Barma Grande; CM = Cro Magnon; GdE = Grotte de Enfants; LaC = La Chapelle; LaF = La Ferrassie; Liu = Liujiang; LQ = La Quina 5; MC = Monte Circeo; MDN = Mai Da Nuoc; Nea = Neandertal; Pet = Petralona; Pred = Predmost; S = Sangiran; Skh = Skhul; UC = Upper Cave-Zhoukoudian; and Zkd = Zhoukoudian ERECT).

The second analysis included MLDG 1704 and 23 other crania, employed eight variables (Table 11), and generated three principal components. For PC 1, the highest loading variables were parietal chord and parietal arc, and these were contrasted mostly with measures of vault width (biparietal breadth and superior facial breadth) (Table 11). For PC 2, frontal chord and frontal arc accounted for most of the variance (Table 11), while PC 3 was mostly explained by maximum frontal breadth (Table 11). A bivariate plot of object scores for PC 1 and PC 2 (Figure 9A) shows PC 1 to separate crania belonging to *H. sapiens* from those of NEAND, *H. heidelbergensis sensu stricto* and ERECT. Specimen MLDG 1704 sits just outside of the convex hull for *H. sapiens*, but clusters close to Cro Magnon 1 and 3 (Figure 9A). A plot of PC 2 versus PC 3 (Figure 9B) shows that these principal components do not discriminate well among taxa. Principal component 3 does, however, distinguish MLDG 1704 from all other crania, with its high positive score. For PC 3, its score is outside of the range of all crania, exceeding the next highest score by 0.29 eigenfactor units (almost double the difference between the *H. sapiens* and NEAND means). *Z*-tests of object scores indicate that the difference between LL 1 and the *H. sapiens* mean is not significant for all PCs [PC 1 *z*0.75; PC 2 *z*1.51; approaching significance for PC 3 (*z*1.62, *p*0.06)]. In contrast, the mean difference for NEAND is significant for PC 1 (*z*-6.89, *p*0.001), but not for PC 2 (*z*1.70) or PC 3 (*z*1.22).

**Figure 10:**
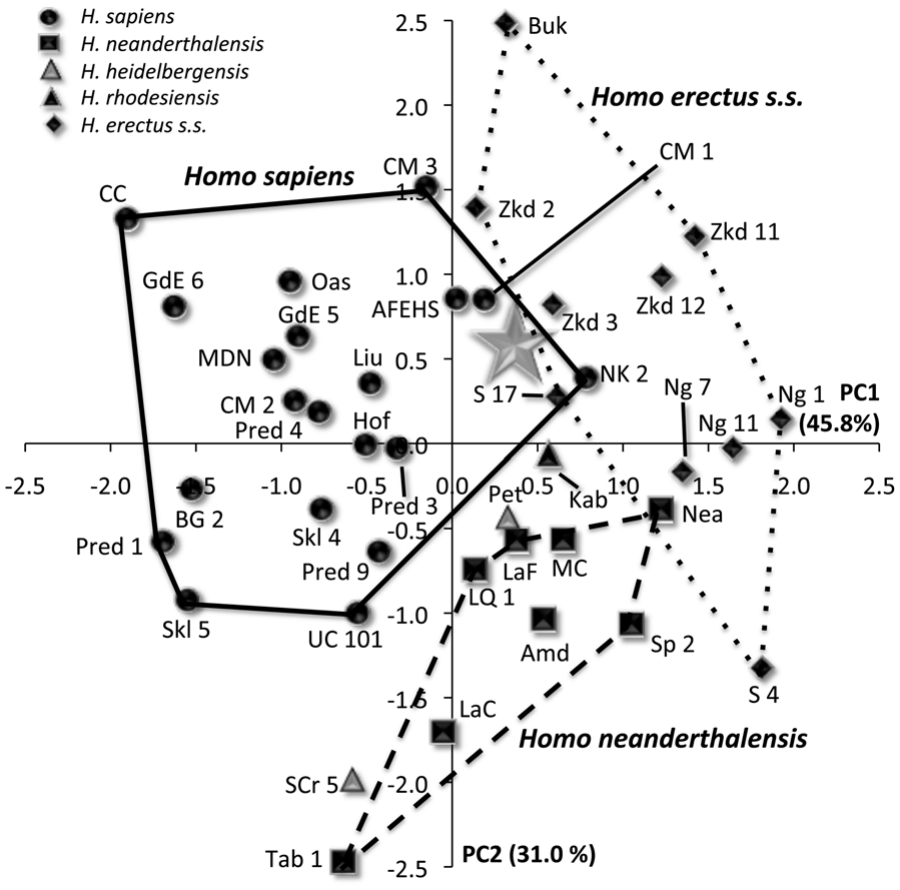
Object plot from principal components analysis including MLDG 1704 and 43 later Pleistocene fossil crania. NB: Gray star = MLDG 1704; AFEHS = African early *H. sapiens*; Amd = Amud; BG = Barma Grande; Buk = Buku; CC = Combe Capelle; CM = Cro Magnon; Kei = Keilor; Hof = Hofmeyr; GdE = Grotte de Enfants; Kab = Kabwe; LaC = La Chapelle; LaF = La Ferrassie; Liu = Liujiang; LQ = La Quina 5; MC = Monte Circeo; MDN = Mai Da Nuoc; Mld = Mladec; Nea = Neandertal; Ng = Ngandong; NK = Nazlet Khater 2; Oas = Oase; Pet = Petralona; Pred = Predmost; S = Sangiran; SCr = Sima de Los Huesos cranium; Skh = Skhul; Sp = Spy; Tab = Tabun; UC = Upper Cave-Zhoukoudian; and Zkd = Zhoukoudian ERECT.

The final analysis included MLDG 1704 and 43 other crania, employed 6 variables (Table 11), and generated two principal components. For PC 1, parietal chord and parietal arc explained most of the variance (Table 11). For PC 2, frontal arc was contrasted with maximum frontal breadth (Table 11). A bivariate plot of object scores for PC 1 and PC 2 (Figure 10) shows good separation between *H. sapiens* on the one hand, and NEAND and ERECT on the other. Specimen MLDG 1704 sits just within the *H. sapiens* convex hull, but near the edge of the ERECT range. It also sits close to the Nazlet Khater 2 cranium from Egypt, a late Pleistocene specimen also possessing a mix of modern and archaic characters [28]. The object score of MLDG 1704 for PC 1 is closest to the archaic Petralona (0.08 eigenfactor units difference) and NEAND Amud 1 (0.13) crania. Moreover, *z*-tests indicate that for PC 1, the difference is significant between MLDG 1704 and the *H. sapiens* mean (excluding Nazlet Khater 2) (*z*2.00, *p*0.03), but not for NEAND (*z*-0.01) or ERECT (*z*-1.04). For PC 2, the difference is significant between MLDG 1704 and NEAND (*z*2.19, *p*0.03), but not for *H. sapiens* (excluding Nazlet Khater 2: *z*0.42) or ERECT (*z*-0.03).

#### Comparison of crania to recent humans

In Tables 12–14 we compare cranial measurements of LL 1 and MLDG 1704 with mixed sex recent humans: East Asian and Eskimo (Table 12), European and African (Table 13) and Australian (Table 14). These samples are compiled from Howells' [29] worldwide dataset (see also, Table 4). Overall, measures of facial width strongly distinguish LL 1 from recent humans, with its very broad face. Bizygomatic breadth (c144 mm) contrasts strongly with recent human means (125–135 mm; *z*1.49–3.16, *p*0.06-0.0008). Bijugal breadth (c134 mm) is similarly enlarged (recent human means 113–117 mm; *z*2.57–4.19, *p*0.005-0.00001). Its bimaxillary breadth (c108 mm) contrasts strongly with recent sample means (92–97 mm; *z*1.83–3.19, *p*0.03-0.0007). Bifrontal breadth (c106 mm) is greatly enlarged (96–100 mm; *z*2.00-1.50, *p*0.06-0.02). Biorbital chord (c114 mm) also contrasts strongly with recent humans (97–99 mm; *z*3.40–5.66, *p*0.001-<0.00001), its value being outside of the range of all samples. Finally, interorbital breadth in LL 1 (25 mm) is also broad compared with recent populations (means 18–23 mm; *z*1.00–3.48, *p*0.15-0.0003).

**Table 12:** Comparison of LL 1 and MLDG 1704 with Howells [29] recent East Asian and Eskimo samples (significant *z*-scores in bold).¶

Howells	Fossil	E. Asian			Eskimo		
Measurement (Abbrev.)	Value	(n = 681)			(n = 108)		
		*μ±σ*	*z*	*p*	*μ±σ*	*z*	*p*
*LL 1*							
Bistephanic breadth (STB)	103	112±7	−1.28	0.09	101±7	0.28	0.38
Bizygomatic breadth (ZYB)	[144]	132±8	1.50	0.06	135±6	1.49	0.06
Nasion-prosthion height (NPH)	64	67±6	−0.50	0.30	69±4	−1.24	0.10
Facial index (NPH/ZYB)	44	51±3	**−2.33**	**0.01**	51±3	**−2.32**	**0.01**
Nasal height (NLH)	47	51±4	−1.50	0.06	52±3	−1.66	0.05
Bijugal breadth [JUB]	[134]	116±7	**2.57**	**0.005**	117±6	**2.82**	**0.002**
Orbit height (OBH)	34	34±2	n.a.	n.a.	36±2	−1.00	0.16
Orbit breadth (OBB)	44	39±2	**2.50**	**0.006**	41±2	1.49	0.06
Orbit index (OBH/OBB)	76	87±5	**−2.20**	**0.01**	87±5	**−2.19**	**0.01**
Nasal breadth (NLB)	[32]	27±2	**2.50**	**0.006**	24±2	**3.98**	**0.00006**
Nasal index (NLB/NLH)	71	52±5	**3.80**	**0.00005**	45±4	**6.47**	**<0.00001**
Bimaxillary breadth (ZMB)	[108]	97±6	**1.83**	**0.03**	96±6	**1.99**	**0.02**
Bifrontal breadth (FMB)	[106]	96±5	**2.00**	**0.02**	97±4	**2.24**	**0.01**
Biorbital breadth (EKB)	[114]	97±5	**3.40**	**0.0003**	98±4	**3.98**	**0.00006**
Interorbital breadth (DKB)	25	21±2	**2.00**	**0.02**	18±2	**3.48**	**0.0003**
Simotic chord (WNB)	4	8±2	**−2.00**	**0.02**	6±2	−1.00	0.16
Malar length, inferior (IML)	(26)	35±4	**1.67**	**0.04**	40±4	**−3.48**	**0.0003**
Supraorbital projection (SOS)	6	6±1	n.a.	n.a.	5±1	1.00	0.16
Malar subtense (MLS)	8	12±2	**−2.00**	**0.02**	12±2	**−1.99**	**0.02**
Glabella projection (GLS)	4	3±1	1.00	0.15	3±1	1.00	0.16
Frontal chord (FRC)	112	110±5	0.60	0.27	111±5	0.20	0.42
Frontal subtense (FRS)	30	26±3	1.33	0.09	27±3	1.00	0.15
Frontal curvature index (FRS/FRC)	27	23±2	**2.00**	**0.02**	24±2	1.49	0.06
*MLDG 1704*							
Bistephanic breadth (STB)	114	112±7	0.29	0.38	101±7	**1.85**	**0.03**
Maximum frontal breadth (XFB)	125	115±7	1.43	0.07	110±4	**3.73**	**0.0001**
Bifrontal breadth (FMB)	107	96±5	**2.20**	**0.01**	97±4	**2.49**	**0.007**
Frontal chord (FRC)	116	110±5	1.40	0.08	111±5	1.00	0.16
Parietal chord (PAC)	107	110±7	−0.43	0.33	113±6	−1.00	0.16
Parietal/frontal chord index (PAC/FRC)	92	101±6	−1.50	0.06	102±6	−1.66	0.05

¶ Fossil values in round brackets are estimates, values in square brackets estimated by measuring to the midline and doubling; sample compositions see Table 4; descriptive statistics for Howells' samples calculated by us from raw data; *z*-test results do not employ Bonferroni correction as per [56].

**Table 13 pone-0031918-t013:** Comparison of LL 1 and MLDG 1704 with Howells [29] recent European and African samples (significant *z*-scores in bold).¶

Howells	Fossil	European			African		
Measurement (Abbrev.)	Value	(n = 317)			(n = 365)		
		*μ±σ*	*z*	*p*	*μ±σ*	*z*	*p*
*LL 1*							
Bistephanic breadth (STB)	103	116±7	**−1.85**	**0.03**	108±7	0.71	0.23
Bizygomatic breadth (ZYB)	[144]	130±6	**2.33**	**0.01**	125±6	**3.16**	**0.0008**
Nasion-prosthion height (NPH)	64	66±5	−0.40	0.34	62±6	0.33	0.36
Facial index (NPH/ZYB)	44	51±3	**−2.33**	**0.01**	50±4	−1.50	0.06
Nasal height (NLH)	47	50±3	1.00	0.15	47±4	n.a.	n.a.
Bijugal breadth [JUB]	[134]	114±5	**3.99**	**0.00004**	113±5	**4.19**	**0.00001**
Orbit height (OBH)	34	33±2	0.50	0.30	33±2	0.50	0.30
Orbit breadth (OBB)	44	39±2	**2.50**	**0.006**	39±2	**2.50**	**0.006**
Orbit index (OBH/OBB)	76	66±9	1.11	0.13	84±6	−1.33	0.09
Nasal breadth (NLB)	[32]	25±2	**3.49**	**0.0002**	28±2	**2.00**	**0.02**
Nasal index (NLB/NLH)	71	50±4	**5.24**	**<0.00001**	59±5	**2.40**	**0.008**
Bimaxillary breadth (ZMB)	[108]	92±5	**3.19**	**0.0007**	94±5	**2.80**	**0.002**
Bifrontal breadth (FMB)	[106]	97±4	**2.25**	**0.01**	98±4	**2.00**	**0.02**
Biorbital breadth (EKB)	[114]	97±3	**5.66**	**<0.00001**	97±4	**4.24**	**0.0001**
Interorbital breadth (DKB)	25	22±2	1.50	0.06	23±2	1.00	0.15
Simotic chord (WNB)	4	9±2	**−2.50**	**0.006**	9±3	**−1.66**	**0.04**
Malar length, inferior (IML)	(26)	35±3	**1.66**	**0.04**	36±4	1.00	0.15
Supraorbital projection (SOS)	6	6±1	n.a.	n.a.	6±1	n.a.	n.a.
Malar subtense (MLS)	8	10±2	1.00	0.15	11±2	−1.50	0.06
Glabella projection (GLS)	4	3±1	1.00	0.15	2±1	**2.00**	**0.02**
Frontal chord (FRC)	112	110±5	0.40	0.34	108±5	0.80	0.21
Frontal subtense (FRS)	30	26±3	1.33	0.09	27±3	1.00	0.15
Frontal curvature index (FRS/FRC)	27	23±2	**2.00**	**0.02**	25±2	1.00	0.15
*MLDG 1704*							
Bistephanic breadth (STB)	114	116±7	−0.29	0.38	108±7	0.89	0.19
Maximum frontal breadth (XFB)	125	119±6	1.00	0.15	111±6	**2.33**	**0.01**
Bifrontal breadth (FMB)	107	97±4	**2.50**	**0.006**	98±4	**2.25**	**0.01**
Frontal chord (FRC)	116	110±5	1.20	0.11	108±5	1.60	0.05
Parietal chord (PAC)	107	111±6	−0.67	0.25	111±6	−0.67	0.25
Parietal/frontal chord index (PAC/FRC)	92	101±6	−1.50	0.06	98±6	−1.00	0.15

¶ Fossil values in round brackets are estimates, values in square brackets estimated by measuring to the midline and doubling; sample compositions see Table 4; descriptive statistics for Howells' samples calculated by us from raw data; *z*-test results do not employ Bonferroni correction as per [56].

**Table 14:** Comparison of LL 1 and MLDG 1704 with Howells [29] recent Australian samples (significant *z*-scores in bold).¶

Howells	Fossil	Australian		
Measurement (Abbrev.)	Value	(n = 165)		
		*μ±σ*	*z*	*p*
*LL 1*				
Bistephanic breadth (STB)	103	102±7	0.14	0.44
Bizygomatic breadth (ZYB)	[144]	131±7	**1.85**	**0.03**
Nasion-prosthion height (NPH)	64	62±5	0.40	0.34
Facial index (NPH/ZYB)	44	47±3	−1.00	0.16
Nasal height (NLH)	47	48±3	−0.33	0.37
Bijugal breadth [JUB]	[134]	116±6	**2.99**	**0.001**
Orbit height (OBH)	34	32±2	1.00	0.16
Orbit breadth (OBB)	44	41±2	1.50	0.06
Orbit index (OBH/OBB)	76	80±6	−0.66	0.25
Nasal breadth (NLB)	[32]	27±2	**2.49**	**0.006**
Nasal index (NLB/NLH)	71	58±5	**2.59**	**0.005**
Bimaxillary breadth (ZMB)	[108]	94±6	**2.33**	**0.01**
Bifrontal breadth (FMB)	[106]	100±4	1.50	0.06
Biorbital breadth (EKB)	[114]	99±4	**3.74**	**0.0001**
Interorbital breadth (DKB)	25	21±2	**1.99**	**0.02**
Simotic chord (WNB)	4	9±2	**−2.50**	**0.006**
Malar length, inferior (IML)	(26)	39±4	0.25	0.40
Supraorbital projection (SOS)	6	7±1	−1.00	0.16
Malar subtense (MLS)	8	11±2	−1.50	0.06
Glabella projection (GLS)	4	5±1	−1.00	0.16
Frontal chord (FRC)	112	109±5	0.60	0.27
Frontal subtense (FRS)	30	25±2	**2.50**	**0.006**
Frontal curvature index (FRS/FRC)	27	23±2	**1.99**	**0.02**
*MLDG 1704*				
Bistephanic breadth (STB)	114	102±7	**1.71**	**0.04**
Maximum frontal breadth (XFB)	125	109±5	**3.19**	**0.0008**
Bifrontal breadth (FMB)	107	100±4	**−3.50**	**0.0003**
Frontal chord (FRC)	116	109±5	1.40	0.08
Parietal chord (PAC)	107	114±6	−1.16	0.12
Parietal/frontal chord index (PAC/FRC)	92	105±6	−2.16	0.01

¶ Fossil values in round brackets are estimates, values in square brackets estimated by measuring to the midline and doubling; sample compositions see Table 4; descriptive statistics for Howells' samples calculated by us from raw data; *z*-test results do not employ Bonferroni correction as per [56].

Frontal chord length for LL 1 (112 mm) is long, but lies within one standard deviation unit of all samples (means 108–111 mm; *z*0.20–0.80). The value for frontal subtense is, however, high (30 mm), and when this measurement is combined with frontal chord to calculate the frontal curvature index (subtense/chord), it is clear that LL 1 exhibits an exaggerated degree of frontal curvature (27%). This index distinguishes the Chinese fossil from recent humans (means 23–25%; *z*1.00–2.00, *p*0.15–0.02). Glabella projection (4 mm) is well within the range of recent humans except for Africans (non-African: means 3–5 mm, *z*1.00 to -1.00; African: 2±1 mm, *z*2.0, *p*0.02). Supraorbital projection (6 mm) is indistinguishable from recent humans (means 5–7 mm; *z*0.00–1.00/−1.00). Posterior breadth of the frontal bone (STB) is narrow (103 mm) and closely resembles Australian (102±7 mm; *z*0.14) and Eskimo (101±7 mm; *z*0.28) means.

Facial height, or nasion-prosthion height in LL 1 (64 mm), is short, but well within the range of recent humans (means 62–67 mm; *z*0.40 to -0.50). In contrast, its face is short relative to its breadth (facial index = height/bizygomatic breadth) (c44%), distinguishing the specimen from most recent human samples (means 47–51%; *z*-1.00 to -2.33, *p*0.16-0.01). Nasal height (47 mm) is identical to the mean for Africans (47±4 mm) and similar to Australians (48±3 mm), but distinct from all other samples (means 50–51 mm; *z*1.00 to -1.66). The nasal breadth of LL 1 (c32 mm) is broad and significantly different to all recent human sample means (means 24–28 mm; *z*2.00–3.98, *p*0.02-0.00006). The nasal index (breadth/height) (c71%) indicates the nasal skeleton of LL 1 to be unusually broad and highly distinct from recent humans (means 45–59%; *z*2.40–6.47, *p*0.008-<0.00001). The value for orbit height (34 mm) sits well within the range of recent humans (32–36 mm; *z*0.00 to -1.00), while orbit breadth (44 mm) contrasts strongly with them (means 39–41 mm; *z*1.49–2.50, *p*0.06-0.006). Orbit index (height/breadth) reinforces the relatively short (for its breadth) left orbit of LL 1 (76%), its value being significantly different to the sample means for East Asian (87±5%; *z*-2.20, *p*0.01) and Eskimo (87±5; *z*-2.19, *p*0.01). The simotic chord is small in LL 1 (4 mm), emphasising the narrowness of its nasal bones superiorly. Its value strongly distinguishing the specimen from East Asian (8±2 mm; *z*-2.00, *p*0.02), Australian (9±2 mm; *z*-2.50, *p*0.006), European (9±2 mm; *z*-2.50, *p*0.006) and African (9±3 mm; *z*-1.66, *p*0.04) samples.

The inferior length of the zygomatic bone of LL 1 (26 mm) is short and distinguishes the fossil from East Asian (35±4 mm; *z*1.67, *p*0.04), Eskimo (40±4 mm, *z*-3.48, *p*0.0003) and European (35±3 mm; *z*1.66, *p*0.04) means. Malar subtense, providing a measure of projection of the malar at its angle, is low in LL 1 (8 mm), in keeping with its broad and laterally flared zygomatics. Its value is significantly different to means for the East Asian (12±2 mm; *z*-2.00, *p*0.02) and Eskimo (12±2 mm; *z*-1.99, *p*0.02) samples.

Specimen MLDG 1704 can be compared with mean values for five measurements (Tables 12–14). Bistephanic breadth (114 mm) lies within the range of recent humans (means 101–116 mm), but its value is significantly different to the Eskimo (101±7 mm; *z*1.85, *p*0.03) and Australian (102±7 mm; *z*1.71, *p*0.04) means. Maximum frontal breadth (125 mm) is also wide in MLDG 1704 and distinguished from the means of recent humans (means 109–115 mm; *z*1.00–3.73, *p*0.15-0.0008). Bifrontal breadth (107 mm) is characterised by statistically significant values when compared with all recent human samples (means 96–100 mm; *z*2.20 to -3.50, *p*0.01-0.0003). The frontal chord of MLDG 1704 is comparatively long (116 mm), but sits within the range of recent humans (means 108–110 mm; *z*1.00–1.60). The parietal chord is short (107 mm), but sits comfortably within the recent human range (110–114 mm; *z*-0.43 to -1.00). Finally, the ratio parietal/frontal chord in LL 1 is low (92%). Its value is distant from the means of most recent humans samples (non-Australian means 98–102 mm; *z*-1.00 to -1.66, *p*0.15-0.05), being significantly different to the Australian mean (105±6 mm; *z*-2.16, *p*0.01).

#### Virtual endocast

**Figure 11:**
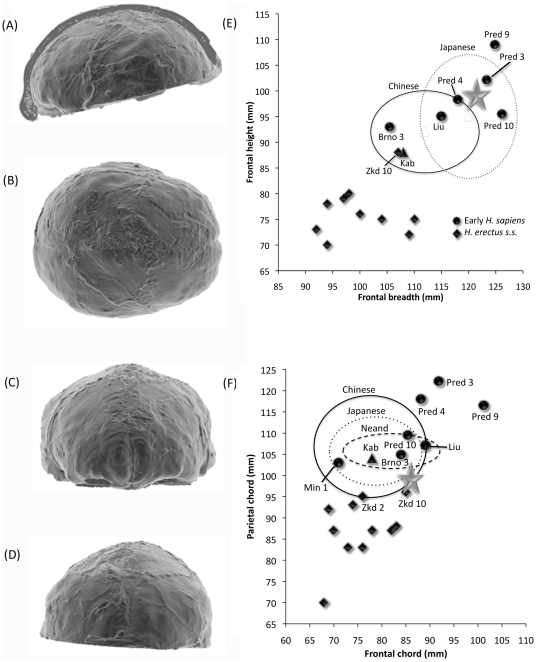
Virtual endocast of MLDG 1704. Left panel: (A) left lateral aspect, (B) superior aspect, (C) anterior aspect, and (D) posterior aspect. Right panel: (E) plot of frontal breadth versus frontal height, and (F) plot of frontal chord versus parietal chord (Gray star = MLDG 1704; ellipses are ranges for samples; Chinese = recent Chinese; Japanese = recent Japanese; Kab = Kabwe; Liu = Liujiang; Min 1 = Minatogawa 1; Nea = Neandertal Pred = Predmost; Zkd = Zhoukoudian).

A 3D virtual endocast was rendered from CT-scans of MLDG 1704 (Figure 11A–D; Figures S1, S2, S3, S4). Measurements of the frontal and parietal lobes were made and are compared in Table 15. They reinforce the visual impression of modern frontal lobes, which are long (86 mm), broad (121 mm) and tall (92 mm), being most like EUEHS (breadth: 120±8 mm, *z*0.11; height: 100±6 mm, *z*-0.15; chord length: 90±7 mm, *z*-0.52). Compared to recent East Asians, its frontal lobe is very long. The Maludong endocast is broader and taller than the Chinese Pleistocene *H. sapiens* cranium from Liujiang (breadth 115 mm, height 95 mm), and much broader than the endocast of the late Upper Pleistocene Japanese Minatogawa I cranium (112 mm). Its frontal lobe is, however, very distinct from the endocast of the *H. rhodesiensis* Kabwe cranium (breadth 108 mm, height 88 mm, chord 78 mm), broader and longer than NEAND endocasts (breadth 107 mm, chord 82 mm) and broader, longer and taller than ERECT (breadth: 99±8 mm; *z*2.64, *p*0.01; height: 74±11 mm; 2.18, *p*0.02; chord: 76±6 mm; *z*1.60).

**Table 15:** Endocast chord measurements (mm) compared (significant *z*-scores in bold).¶

Sample	Frontal	Frontal	Frontal	Parietal
	Breadth	Height	Chord	Chord
MLDG 1704	121	99	86	99
Liujiang	115	95	89	107
Minatogawa 1	112	-	71	103
Kabwe	108	88	78	104
EUEHS	120±8(5)	100±6(5)	90±7(5)	122±7(5)
	(106–126)	(93–109)	(84–101)	(105–122)
*z*-score/*p*	0.11/0.45	−0.15/0.44	−0.52/0.31	**−3.00/0.01**
Recent Chinese	112±5(31)	92±4(31)	79±5(31)	106±4(31)
	(103–122)	(86–100)	(69–89)	(98–114)
*z*-score/*p*	**1.77/0.04**	**1.72/0.04**	1.38/0.08	**−1.72/0.04**
Recent Japanese	120±4(32)	95±5(32)	77±5(32)	107±7(32)
	(112–129)	(83–105)	(64–84)	(95–119)
*z*-score/*p*	0.25/0.40	0.79/0.21	**1.77/0.04**	−1.13/0.13
NEAND	107(6)	-*	82(6)	106(3)
	(103–111)	-*	(72–92)	(104–110)
ERECT	99±8(12)	74±11(12)	76±6(11)	87±7(11)
	(84–110)	(44–88)	(68–85)	(70–96)
*z*-score/*p*	**2.64/0.01**	**2.18/0.02**	1.60/0.07	1.64/0.06

¶ Above the line *μ±σ (n*), below the line (*min.-max.*); *z*-test results do not employ Bonferroni correction as per [56]; data sources see Text S2.

* Data not available because published height for this taxon has been measured from the frontal pole-occipital pole; cannot be measured in MLDG 1704 owing to absence of the occipital bone.

In contrast, the parietal lobes of MLDG 1704 are very short (99 mm), contrasting with the long parietal lobes of EUEHS (122±7 mm; *z*-3.00, *p*0.01), recent Chinese (106±4 mm; *z*-1.72, *p*0.04) and recent Japanese (107±7 mm; *z*-1.13). The parietal chord length for NEAND is also moderate (106 mm), like Liujiang (107 mm) and Minatogawa 1 (103 mm). While the parietal lobes of MLDG 1704 are short, much shorter even than Kabwe (104 mm), they are longer than ERECT (87±7 mm, *z*1.64).

Figure 11E is a bivariate plot of the breadth of the frontal lobes versus frontal height. It confirms the modern size and shape of the frontal of MLDG 1704, its value sitting well within the range of EUEHS (i.e. Predmost crania) and recent Japanese. Figure 11F compares frontal chord and parietal chord dimensions of the endocast and shows MLDG 1704 to be just within the range of recent Chinese, outside of the range of fossil *H. sapiens*, and very close to the ERECT specimen Zhoukoudian 10.

#### Mandibles

Table 16 compares a range of commonly employed mandibular characters and Table 17 body metrics for distinguishing among later Pleistocene hominins. While the symphyseal region of LL 1 has been damaged, in our judgement, it would probably have possessed a chin of Rank 3 [30]. The chin of MLDG 1706 is Rank 3 [30], and while relatively common among Eurasian early *H. sapiens* (29.2–49.5%), the Chinese Tianyaun 1 and Zhirendong 3 mandibles possess Rank 4 chins. Specimens LL 1 and MLDG 1706 lack a vertical keel and lateral tubercles, features which form the major components of the modern human ‘inverted-T’ form chin [31]. In inferior view, the anterior border of the body (beneath the symphysis) is rounded in both LL 1 and MLDG 1706, more like the condition seen in archaic hominins [31]. The mental foramen is located below P_4_/M_1_ in LL 1 and MLDG 1706. Location of this foramen mesial to M_1_ is characteristic of early *H. sapiens* (88–100% presence versus 12.2% NEAND). Mandibular foramen bridging is absent in MLDG 1679, but present in MLDG 1706 (cannot be scored on LL 1). Absence of bridging is common in NEAND (57.1% presence), but rare in Eurasian early *H. sapiens* (0–7% presence).

**Table 16:** Mandibular body traits compared.¶

Sample	Mentum	Mental foramen	Mandibular	Mandibular notch	Retromolar	Medial pterygoid
	osseum rank	location	foramen bridging	symmetrical	space	tubercle
	(% rank 4)	(% mesial of M_1_)	(% absent)	(% present)	(% absent)	(% absent)
LL 1	?3	P_4_/M_1_	-	-	?Present	-
MLDG 1679	-	-	Absent	Asymmetrical	Present	Absent
MLDG 1706	3	P_4_/M_1_	Present	Asymmetrical	Absent	Absent
Tianyuan 1	4	P_4_/M_1_	Absent	Symmetrical	-	Absent
Zhirendong 3	4	P_4_	-	-	-	-
EAEHS	50.5(4)	90.9(11)	100(3)	100(3)	-	33.3(3)
EUEHS	70.8(4)	92.0(25)	100(16)	100(17)	77.1(17)	90.0(10)
Western EHS	85.7(7)	100(5)	83.3(3)	66.7(3)	60(5)	100(3)
AFEHS	68.8(8)	87.7(7)	100(5)	100(4)	-	100(6)
NEAND	0.0(23)	12.2(31)	42.9(21)	30.8(13)	25(28)	18.8(16)
ERECT ^	-	33(12)	-	-	-	-

¶ Sample abbreviations and compositions see Table 4, except Western EHS ( = MIS3 early modern humans [9]–[10]); data sources [9]–[10] and see Text S2.

^ Most ERECT mandibles possess multiple mental foramina. We have scored a mandible as having a mental foramen mesial to M_1_ only when all foramina are in this position.

**Table 17:** Mandibular body measurements compared (significant *z*-scores in bold).¶

Sample	Anterior symphyseal	Body height at	Body thickness at
	angle	mental foramen	mental foramen
	(°)	(mm)	(mm)
LL 1	-	(28)*	14*
MLDG 1706	∼77	26.9	13.3
Tianyuan 1	∼96	28.7	11.3
Zhirendong 3	91	27.4	16.0
EAEHS	94	29.0,31.0,33.7	12.0,13.0,14.4
EUEHS	96.5±6.2(12)	31.6±4.4(12)	12.4±1.4(11)
*Z*-score/*p* LL 1	-	−0.79/0.22	1.09/0.14
*Z*-score/*p* MLDG 1706	**−3.02/0.005**	-	0.62/0.27
Western EHS	89,91	27.5,33.2,35.3	11.6,12.2,15.7
AFEHS	86.4±6.4(5)	35.0,36.0,40.5	13.2,15.0,16.6
*Z*-score/*p* MLDG 1706	−1.34/0.12	-	-
NEAND	80.8±7.3§(18)	32.3±3.6(26)	15.5±1.8§(26)
*Z*-score/*p* LL 1	-	−1.17/0.12	−0.82/0.21
*Z*-score/*p* MLDG 1706	−0.51/0.30	-	−1.20/0.12
ERECT	68.9±12.7(6)	-	-
*Z*-score/*p* MLDG 1706	0.59/0.29	-	-

¶ Sample abbreviations and compositions see Table 4, except Western EHS ( = MIS3 early modern humans [9]–[10]); data sources [9]–[10] and see Text S2; *μ±σ(n)*; *z*-test results do not employ Bonferroni correction as per [56].

* Value taken slightly distal to mental foramen owing to damage.

§ *T*-test EUEHS and NEAND mean difference: one-tailed *p*<0.05-0.01.

Both Maludong mandibles show asymmetry of the mandibular notch. However, the coronoid process of MLDG 1679 is disproportionately large, a feature common among NEAND, while in MLDG 1706 it is greatly reduced, the *H. sapiens* condition. In LL 1, the coronoid process is large, but its proportions cannot be assessed owing to an absence of the notch and condylar process. Specimens LL 1 and MLDG 1679 possess a retromolar space (M_3_ is uncovered [21]), a common characteristic of NEAND (presence: 75%, versus 32.9–40% in early *H. sapiens*). In MLDG 1706, the M_3_ is partially covered; scored here as absence of a retromolar space. While the medial pterygoid attachment area is strongly scarred in both Maludong mandibles, they lack a prominent superior pterygoid tubercle (present: NEAND 81.2%, Eurasian early *H. sapiens* 10–76.7%). Finally, the crest of the mandibular notch meets the condyle laterally in MLDG 1679, but it is more medially located in MLDG 1706. Medial placement of the crest is found frequently in NEAND (63% presence, versus 100% absence in Western and European *H. sapiens*) and characterises the Dar-es-Soltane 5 mandible with its apparent archaic affinities [1], [32].

Internally, the alveolar plane of LL 1 and MLDG 1706 is posteriorly inclined and the transverse tori are thickened. This is a common feature among archaic later Pleistocene hominins such as Témara 1 (North Africa), but is largely absent from early *H. sapiens* [32]. Externally, the symphysis is somewhat undercut in lateral aspect, and its anterior symphyseal angle is low (77°), a value closest to NEAND (80.8±7.3°; *z*-0.51) and the Témara 1 mandible (80°). In contrast, Pleistocene *H. sapiens* angles are more acute (means 86.6–96.5°; *z*-1.34 to -3.02), as seen also in the East Asian mandibles Tianyuan 1 (∼96°) and Zhirendong 3 (91°). Body height (26.9 mm) and thickness (13.3 mm) at the level of the mental foramen in MLDG 1706 are comparatively low, showing the specimen to be similar to modern humans in its size. Its body height sits just outside of the range of Pleistocene East Asian *H. sapiens* mandibles (range 27.4–33.7 mm), but body thickness is comfortably within their range (11.3–14.4 mm). Body height (28 mm) and thickness (14 mm) measured slightly posterior to the mental foramen in LL 1 is similar to the Maludong specimen (Table 16).

#### Dentition

The mostly well preserved, but worn, dental crowns of LL 1 and MLDG 1706 (Figure 6–7), and an isolated maxillary third molar (MLDG 1747: Figure 12), also reveal important information about their morphology and affinities. Buccolingual (BL) crown diameters and descriptive statistics for comparative samples are provided in Table 18 (mandibular dentition) and Table 19 (maxillary dentition).

**Figure 12:**
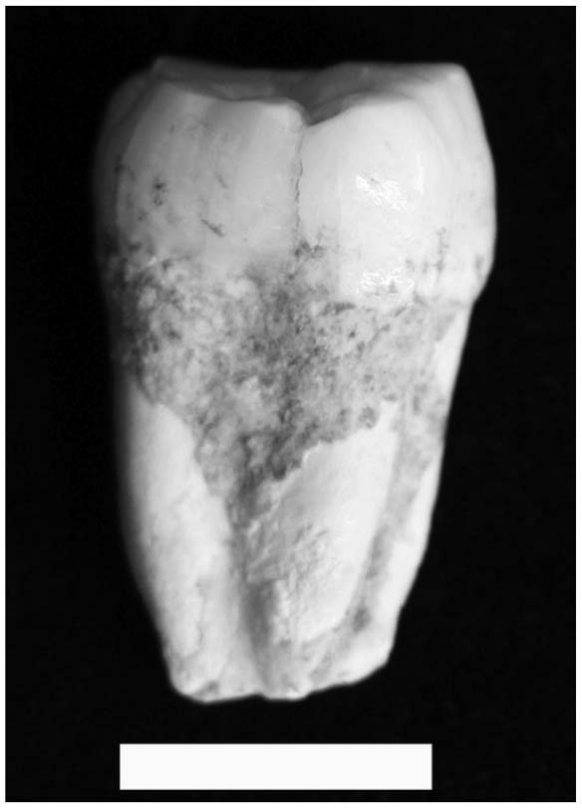
Isolated M^3^ – specimen MLDG 1747 (scale bar = 1 cm) exhibiting marked taurodontism.

**Table 18:** Comparison of mandibular dental crown buccolingual diameters (mm) (significant *z*-scores in bold).¶

Fossil/Sample	I2	C	P3	M2	M3
LL 1	6.7	8.4/7.6§	9.3/9.5§	-	10.7/10.3§
MLDG 1679	-	-	-	11.9	11.6
Tianyuan 1	7.1	8.9	8.2	10.7	11.3
Zhirendong 1, 2	-	-	-	10.3	10.1, 0.3
Eastern EMH	6.9±0.3‡(6)	8.3±0.5†(6)	8.4±0.1†(5)	11.1±0.4†(7)	10.4±0.4†(7)
*z*-score/*p* LL 1	−0.64/0.28	−0.56/0.30	**−9.13/0.0003**	-	−0.23/0.41
*z*-score/*p* MLDG 1679	-	-	-	1.87/0.05	**2.81/0.01**
Western MUP†	6.8±0.5(22)	8.6±0.7(19)	8.5±0.5(18)	11.0±0.8(28)	10.8±0.9(17)
*z*-score/*p* LL 1	−0.20/0.42	−0.08/0.46	**−1.75/0.04**	-	−0.88/0.19
*z*-score/*p* MLDG 1679	-	-	-	1.11/0.13	0.86/0.20
Western EMH†	7.2±0.5(5)	8.7±0.8(6)	8.4,9.0	11.2±1.1(7)	10.0,11.7,14.2
*z*-score/*p* LL 1	−0.91/0.20	−0.81/0.22	-	-	-
*z*-score/*p* MLDG 1679	-	-	-	0.60/0.28	-
Middle Palaeolithic MH†	7.2±0.5(10)	8.3±0.8(10)	8.8±0.5(8)	11.0±0.7(10)	10.8±0.6(8)
*z*-score/*p* LL 1	−0.9/0.18	−0.36/0.36	1.13/0.14	-	−0.88/0.19
*z*-score/*p* MLDG 1679	-	-	-	1.23/0.12	1.26/0.12
Neandertals†	7.7±0.5(28)	8.9±0.7(33)	9.0±0.7(33)	11.0±0.7(36)	11.0±0.8(42)
*z*-score/*p* LL 1	**−1.97/0.02**	−1.27/0.10	0.56/0.28	-	−0.88/0.19
*z*-score/*p* MLDG 1679	-	-	-	1.27/0.10	0.74/0.23
ERECT‡	7.1±0.5(11)	9.1±0.7(11)	10.0±0.6(16)	12.8±0.9(18)	11.5±1.1(14)
*z*-score/*p* LL 1	−0.77/0.23	−1.05/0.08	−0.97/0.17	-	−0.88/0.19
*z*-score/*p* MLDG 1679	-	-	-	−0.97/0.17	−0.09/0.46

¶ Sample abbreviations and compositions see Table 4 and [9]–[10]; *μ±σ(n)*; *z*-test results do not employ Bonferroni correction as per [56].

† Comparative samples from Shang et al. [9].

‡ Data compiled by the authors from literature (see Table 4 and Text S2).

§ Mean of left and right used in *z*-test.

**Table 19:** Comparison of maxillary dental crown buccolingual diameters (mm) (significant *z*-scores in bold).¶

Fossil/Sample	P4	M1	M3
LL 1	11.0	11.7	-
MLDG 1747	-	-	12.5
EAEHS‡	10.5(4)	12.3±0.7(8)	11.7±1.3(5)
*z*-score/*p* LL 1	−0.49/0.31	−0.81/0.22	-
*z*-score/*p* MLDG 1747	-	-	0.56/0.30
Early Upper Palaeolithic†	-	-	11.6±1.1(27)
*z*-score/*p* MLDG 1747	-	-	0.80/0.21
Middle Palaeolithic HS†	-	-	11.9(1)
Neandertals†	10.0±0.8(21)	12.0±0.8(34)	11.9±1.4(29)
*z*-score/*p* LL 1	1.40/0.08	−0.37/0.35	-
*z*-score/*p* MLDG 1747	-	-	0.42/0.33
Qafzeh-Skhul†	10.2±0.8(10)	12.2±0.7(18)	11.7±0.6(6)
*z*-score/*p* LL 1	0.95/0.18	−0.70/0.24	-
*z*-score/*p* MLDG 1747	-	-	0.42/0.33
Early Upper Palaeolithic HS†	9.9±0.6(25)	12.2±0.8(37)	11.6±1.1(27)
*z*-score/*p* LL 1	**1.80/0.04**	−0.62/0.27	-
*z*-score/*p* MLDG 1747	-	-	0.80/0.21
ERECT‡	11.5±1.0(16)	13.0±1.0(11)	11.8±0.9(8)
*z*-score/*p* LL 1	−0.49/0.31	−1.24/0.12	-
*z*-score/*p* MLDG 1747	-	-	0.73/0.24

¶ Sample abbreviations and compositions see Table 4 and [58]–[59]; *μ±σ(n)*; *z*-test results do not employ Bonferroni correction as per [56].

‡ Data compiled by the authors from literature (see Table 4 and Text S2).

† Comparative sample statistics from Trinkaus et al. [58]–[59].

The LL 1 I_2_ crown (6.7 mm) is narrow, its value sitting well within the range of comparative samples except NEAND with their broad incisors (*H. sapiens* means 6.9–7.2 mm; *z*-0.20 to -0.95; NEAND 7.7±0.5; *z*-1.97, *p*0.02; ERECT 7.1±0.5 mm; *z*-0.77). The mandibular canine crowns of LL 1 are also small (8.4/7.6 mm), and while not significantly different to any comparative sample mean, its BL diameter is closest to EAEHS (8.3±0.5 mm; *z*-0.56) and a Middle Palaeolithic *H. sapiens* sample (8.3±0.8 mm; *z*-0.36). In contrast, its P_3_ BL diameters (9.3/9.4 mm) are large like NEAND (9.0±0.7; *z*0.56) and ERECT (10.0±0.6 mm; z-0.97). Its P_3_ crown width is significantly different to mean values for EAEHS (8.4±0.1 mm; *z*-9.13, *p*0.0003) and a Western Middle Upper Palaeolithic Human sample (8.5±0.5; *z*-1.75, *p*0.04). The M_2_ crown of MLDG 1679 is broad (11.9 mm), but its value is not significantly different to comparative sample means (11.0–12.8 mm; although it is approaching significance for EAEHS *z*1.87, *p*0.05). Mandibular M_3_ crown BL diameters for LL 1 (10.7/10.3 mm) and MLDG 1679 (11.6 mm) are moderate to large. The crown of the former specimen is not significantly larger than comparative means (10.4–11.5 mm; *z*-0.23 to -0.88), while the latter is distinct from EAEHS (10.4±0.4 mm; *z*2.81, *p*0.01).

The measurable maxillary crowns of LL 1 are comparatively broad. Its P^4^ BL (11.0 mm) is most like ERECT (11.5±1.0 mm; *z*-0.49, *p*0.31) and is distinct from *H. sapiens* (Qafzeh-Skhul 10.2±0.8 mm; *z*0.95; Upper Palaeolithic *H. sapiens* 9.9±0.6 mm; *z*1.80, *p*0.04) and NEAND (10.0±0.7 mm; *z*1.40). In contrast, its M^1^ crown is narrow (11.7 mm), and while its value is well within the range of all comparative samples, it is closest to the NEAND mean (12.0±0.8; *z*-037). The BL diameter of an isolated M^3^ MLDG 1747 (12.5 mm) is comparatively large, but sits within the range of all samples listed in Table 19, being equally close to the Qafzeh-Skhul (11.7±0.6; *z*0.42) and NEAND (11.9±1.4; *z*0.42) means.

Measurements made on CT-scans of the *in situ* M_3_ of MLDG 1679 (not given) indicate that this tooth is taurodont (Taurodontism index [33] 26.1%, or hypotaurodont). Additionally, MLDG 1747 is also taurodont (Figure 12), its three roots being fused for most of their course. Taurodontism is rare among recent and EUEHS humans [34]–[35], but is commonly considered a distinguishing feature of NEAND [35]–[36].

## Discussion

The partial human skull from Longlin Cave and the human calotte, partial mandibles and teeth from Maludong both present a range of individual features and a composite of characters not seen among Pleistocene or recent populations of *H. sapiens*. It is clear that they share no particular affinity with either Pleistocene East Asians, such as Liujiang or Upper Cave 101, or recent East Asians. These features belong to multiple developmental-functional complexes [37], spanning the neurocranial vault, including endocranium, cranial base, facial skeleton, mandible and dentition. Where they can be assessed, metrical dimensions involved are characterised mostly by moderate to high heritability [38]–[41]. Given their morphological similarity, close geographical proximity (<300 km apart) and young geological age (i.e., Pleistocene-Holocene transition), it seems likely that both samples belong to the same population.

Multivariate analysis of vault shape, a method shown to track neutral genetic distances [42], indicates a somewhat mixed picture with respect to the phenetic affinities of LL 1 and MLDG 1704. The dominant phenetic signal in these analyses, as indicated by the first principal component (accounting for 45–46% of total variance), shows LL 1 and MLDG 1704 to be at the edge of variation within Pleistocene *H. sapiens*, and in some analyses, on the edge also of *H. erectus* variability. A weaker phenetic signal, revealed particularly by principal component 3 (∼12–14% of total variance), shows them to exhibit a unique cranial shape among all later Pleistocene hominins.

A range of features support the conclusion that these remains show affinities to *H. sapiens*:

- **Neurocranium**: moderately projecting and laterally thin supraorbital part, which has the bipartite form in MLDG 1704; frontal bone with a moderate chord and arc length, but broad maximum width; and an endocast with long, broad and tall frontal lobes.
- **Viscerocranium**: narrow superior facial breadth; vertically short facial skeleton (superior facial height, orbit height and nasal height); and moderate nasal breadth relative to height.
- **Mandible**: mesial position of the mental foramen; and absence of a medial pterygoid tubercle.
- **Dentiton**: small (narrow) anterior dental crowns.

At the same time, the Longlin and Maludong fossils possess many features that are either rare or absent among Pleistocene and recent *H. sapiens*, many of them being putative plesiomorphies of later *Homo*. These include:

- **Neurocranium**: moderate endocranial volume; highly arched frontal squama; short parietal bones; endocast with short parietal lobes; narrow postorbital region; and absence of a bipartite supraorbital morphology in LL 1.
- **Cranial base** (LL 1 only): a mandibular fossa that is long (A-P), broad (M-L) and deep (S-I).
- **Viscerocranium** (LL 1 only): strong alveolar prognathism; flat mid-face, both at the nasal root and aperture and zygomatic process of the maxilla; broad facial skeleton (interorbital, bizygomatic and bimaxillary); very narrow nasal bones; broad piriform aperture; absence of a canine fossa, and possessing a deep *sulcus maxillaris*; zygomatic arch is laterally flared; zygomatic strongly angled such that its inferior margin sits well lateral to the superior part; zygomatic tubercle is small and sits lateral to a line project from the orbital pillar (anterior aspect); the anterior masseter attachment area is marked by a broad and deep sulcus; strong transverse incurvation of lateral orbital pillar (lateral aspect); and anterior placement of the anterior wall of the zygomaticoalveolar root (above P^4^/M^1^).
- **Mandible**: absence of a sagittal keel and distinct lateral tubercles; small chin (MLDG 1706 Rank 3, LL 1 Rank ?3); mandibular foramen bridging (MLDG 1706); thickened transverse tori; asymmetrical mandibular notch (MLDG 1679); retromolar space; crest of the mandibular notch positioned laterally (MLDG 1679); and a low anterior symphyseal angle (MLDG 1706).
- **Dentition**: broad post-canine crowns (large BL diameters); and taurodont molars.

The finding of human remains with such a combination of modern (*H. sapiens*) and archaic (putative plesiomorphic) characters is unusual, especially in Eurasia. In Africa, there are several Pleistocene remains that also combine modern features with putative later *Homo* plesiomorphies: from Klasies River Mouth Cave [43]–[44] and Hofmeyr [45] (South Africa), Iwo Eleru (Nigeria) [46], Nazlet Khater (Egypt), and Dar-es-Soltane and Témara (Morocco) [1], [28], [45]. Most of them are, however, much older than Longlin and Maludong: Dar-es-Soltane and Témara are undated, but they were associated with the Aterian lithic assemblage which has recently been dated to between 107±3 ka and 96±4 ka at another Moroccan site (La Grotte des Contrebandiers) [47]; the Klasies River Mouth remains are from two units dating >101 ka and >64–104 ka [48]; Nazlet Khater 2 is perhaps ≈42 ka [28]; and Hofmeyr 36.2±3.3 ka [49]. However, the recently described Iwo Eleru calvaria has been dated ∼16.3-11.7 ka [46] and is clearly of similar age to the Chinese remains.

Various Upper Pleistocene fossils outside of Africa have also been described as exhibiting an unusual mosaic of characters [e.g. 28]. Some of them, such as from Skhul and Qafzeh (Israel) and Pestera cu Oase (Romania) have been included in our analyses, and overall seem to be metrically well within the range of Pleistocene *H. sapiens* (e.g. Figures 8–9). The former (Levantine) samples do, however, show some similarities to LL 1 and MLDG 1704 in univariate comparisons.

How might the presence of this unusual morphology during the Pleistocene-Holocene transition of East Asia be explained? The remains from Longlin and Maludong could represent very robust individuals within a previously unknown Epipalaeolithic population in southwest China. We consider this to be an unsatisfactory explanation because of the presence of several apparently unique features combined with an unusual mixture of modern and archaic features is seen in several specimens and spans multiple developmental-functional complexes (as noted above). Moreover, this hypothesis could also be invoked to explain the morphology of remains from Klasies River Mouth Cave, Hofmeyr, Iwo Eleru, Nazlet Khater, Dar-es-Soltane, Témara and Zhirendong, but has not because many of their archaic features are rare or absent among *H. sapiens*. The same situation applies to the Longlin and Maludong remains, as shown strongly here.

In our opinion, there are more plausible explanations. One possibility is that the Longlin and Maludong remains represent a late surviving archaic population, perhaps similar to that sampled at Dar-es-Soltane and Témara [1], [28], [32]. Unfortunately, little is known of the morphology of these North African remains, and their affinities and taxonomy are unclear [1], [28], [32]. Within East Asia, the recently described mandibular fragment from Zhirendong also possesses a mosaic of modern and plesiomorphic characters making its taxonomic status problematic [3], [10]–[11]. It has, although, been dated on stratigraphic grounds to >100 ka [10], similar in age to the North African Aterian assemblage, but much older than Longlin and Maludong. Another recently described specimen from the site of Salkhit (Mongolia) has also been described as belonging to an unspecified archaic taxon [50]. Dating is uncertain, although, a preliminary date of ∼20 ka has apparently been reported [3]. Moreover, doubts about its archaic affinities have been expressed [3] (Note: we have been unable to include this specimen in our analyses as we found errors in the measurements of this and other specimens included in Table 1 of Coppens et al. [50]).

Another possible explanation is that the unusual morphology of the Longlin and Maludong remains results from the retention of a large number of ancestral polymorphisms in a population of *H. sapiens*. The concept of incomplete lineage sorting is commonly invoked to explain morphologically mixed groups where the features of interest are present also in allopatric populations belonging to the same taxon [51]. Related to this, recent morphological studies have suggested that Pleistocene *H. sapiens* was deeply geographically subdivided within Africa prior to its dispersal into Eurasia [52]. This explanation has also been invoked to explain the unusual morphology of the Iwo Eleru calvaria [46]. The morphology documented at Longlin and Maludong might be interpreted as consistent with this hypothesis, the Chinese remains perhaps sampling a previously unknown human population (or migration?) that may not have contributed genetically to recent East Asians. Ancient DNA could allow for a test of this idea, however, our ongoing attempts to extract DNA from a specimen from Maludong have so far proven unsuccessful owing to a lack of recoverable genetic material.

Either way, the presence of the unusual morphology sampled at Longlin and Maludong during the Pleistocene-Holocene transition indicates that the evolutionary history of humans in East Asia is more complex than has been understood until now. It further highlights the need for much more research in the region as a matter of priority.

## Methods

### Radiocarbon dating

Fifteen charcoal samples for AMS radiocarbon assay were prepared and measured at the ANTARES-STAR Accelerator Mass Spectrometry Facility at the Australian Nuclear Science and Technology Organisation described in [60]. All samples were pre-treated and converted to graphite following methods described by [61]. The external surfaces of charcoal pieces selected for assay were scraped with a cleaned scalpel to remove sediment and soil attached to charcoals. The samples were then cut into smaller pieces to increase surface areas for more efficient chemical pre-treatment. Each sample was then treated with an acid-base-acid sequence as follows:

- 2 M HCl at 60°C for 2 hours to remove carbonate and any infiltrated fulvic acid contaminants,
- 0.5–4% NaOH at 60°C for 10 hours to remove infiltrated fulvic and humic acid contaminants. This treatment is commenced with a very weak alkali solution of 0.5% NaOH then with successively stronger solutions until the solution is clear or until all humic acids are removed,
- 2 M HCl at room temperature for 4 hours to remove any atmospheric CO_2_, which was absorbed by the samples during the alkali treatment.

The cleaned charcoal pieces are finally placed into an oven at 60°C for 2–3 days to dry and then taken for combustion using routine methods for conversion of charcoal to graphite [60]. A portion of each graphite sample was used to determine δ^13^C for mass fractionation correction from the graphitisation process. Measured AMS ^14^C/^13^C ratios are converted to conventional radiocarbon ages after background subtraction and δ^13^C fractional correction. Radiocarbon ages (see Table 1) are given with 1 standard deviation (1σ) precisions ranging from ±0.3 to 0.5. All radiocarbon ages were converted to calibrated calendar ages BP (before-present, 1950) using the CALIB 6.02 calibration software and the IntCal09 data sets [62]. All calendar age errors quoted in this paper are given as 2 standard deviation errors (2σ). Table 1 provides radiocarbon ages and calibrated calendar ages for each charcoal sample measured by AMA. Table S1 provides ancillary information pertaining to sample pretreatment, graphite AMS mass and δ^13^C values used to correct AMS radiocarbon data from Maludong.

### Archaeomagnetics

Detailed theories and methods related to the use of magnetic measurements for reconstructing palaeoclimate and anthropogenic alteration are outlined in [63]–[65]. Bulk sediment samples were taken from every single excavated stratigraphic unit during excavation. These bulk samples were divided into sub-samples, air dried and sieved to remove any large non-magnetic particles (i.e. limestone clasts). The sieved bulk sub-samples were then subjected to a range of mineral magnetic measurements. Low temperature and room temperature dual frequency magnetic susceptibility measurements were undertaken on a Bartington MS2 system. Isothermal remanent magnetisation (IRM) acquisition and backfield curves, hysteresis loops and thermomagnetic curves were run on a Magnetic Measurements Variable field Translation Balance (MM-VFTB).

### Endocast rendering and volume estimation

A virtual endocast of MLDG 1704 was generated from computed tomography (CT) data in *Mimics* (Ver. 13.02) by:

1. Segmenting out extraneous material and generating a mask for MLDG 1704,
2. Generating a cutting plane and converting this mask into a 3D object,
3. Positioning the 3D object such that it closed the open region of the cranium,
4. Generating a mask from the repositioned 3D of the cutting plane,
5. Combining the mask of MLDG 1704 with that of the cutting plane,
6. Using the ‘cavity fill’ tool to create a partial endocast from this combined mask,
7. A 3D surface mesh was then generated from this mask of the endocast and imported into *Strand7* (ver. 2.4), and
8. A solid mesh of the partial endocast was then created in *Strand7* and the volume taken from the model summary.

Six endocasts and their respective volumes were generated from CT scans of complete Holocene age southern African San crania using this same general approach (Figure S1). In these instances ‘holes’ in the masks of the crania representing nerves and blood vessels were filled before applying the ‘cavity fill’ tool to produce the endocasts.

A template of Type I, Type II and Type III [66] landmark points was created to capture the whole surface morphology of the six modern human endocasts (Figure S2). Warping of cranial exterior surface morphology using a mixture of landmark points and slid semi-landmarks has been shown to be highly effective at reproducing target cranial shape [67]. Here we apply a similar methodology, utilising landmarks, pseudo-landmarks and slid semi-landmarks, to these endocrania. The landmark template was designed to capture as much as possible of the endocranial shape that was common to all six modern humans.

Our landmark template consisted of 715 landmarks. We used 33 single points (Type I and II landmarks), 9 curves (the beginning and end of the curves were defined by Type II landmarks, with 8 additional Type III semilandmarks slid between these across the endocranial surface) and 12 polygon regions (9 user defined Type II landmarks with additional slid semilandmarks). The polygon regions were used to capture the morphology of the different lobes of the brain. Four of the polygons were defined by 100 landmarks (9 Type II, 91 slid semilandmarks), with the remaining 8 polygons defined by 25 landmarks (9 Type II, 16 slid semilandmarks). Once the landmarks were placed on all of the crania, Template Optimisation was used to create the ‘mean’ endocranial whole surface shape of these six humans (Figure S3). Template Optimisation has been shown to be accurate in reproducing the target mesh shapes [68].

The mean endocranial shape was registered with NMB 1204 using an Iterative Closest Point (ICP) registration algorithm [69]–[71], to place it in a 3D space relevant to that of the other human endocrania. The modern human endocrania and that of MLDG 1704 were ICP registered with the mean endocranium to minimise any orientation differences between endocranial specimens and the mean shape (Figure S4). STLs of the registered ‘mean’ the San and MLDG 1704 endocasts were imported into *Mimics* and a cutting plane was generated and positioned as above, with the endocast of MLDG 1704 superimposed (Figure S4). This was used to separate that part of the mean San endocast that was not preserved in MLDG 1704. The volume of this separated portion amounted to 39% of the original total brain volume for the mean San endocast. The total brain volume of MLDG 1704 is estimated to be 1327 cm^3^ assuming similarity in proportions between the two.

## Supporting Information

Figure S1 **Endocasts generated from computed tomography.** **a**, MLDG 1704, and San crania: **b**, NMB 4. **c**, NMB 1271. **d**, NMB 1640. **e**, NMB 1204. **f**, NMB 1707. **g**, NMB 1240.
(TIF)

Figure S2 **Varying views of the BW 1204 modern human (San) endocranium specimen showing the landmark and slid semilandmark template as applied to each of the 6 modern human specimens.** Aspect viewed: A) inferior, B) superior, C) frontal, D) ¾ frontal-inferior E) lateral F) ¾ inferior-lateral.
(TIF)

Figure S3 **Landmark template.** A) applied to BW 1204 (as in Figure S2), B) same landmark template applied to specimen BW 1240, C) and mean modern (San) endocranial shape generated from the average landmark configuration of the 6 modern human specimens.
(TIF)

Figure S4 **Registered mean San (dark gray) and MLDG 1704 (light gray) endocasts superimposed showing cutting plane.**
(TIF)

Table S1 **Radiocarbon data for Maludong.**
(DOCX)

Text S1 **Archaeomagnetics - results.**
(DOCX)

Text S2 **Additional references: sources of metrical and morphological data and dating estimates.**
(DOCX)
