# Supplementary figures and images for: Human Remains from the Pleistocene-Holocene Transition of Southwest China Suggest a Complex Evolutionary History for East Asians
Source: PLoS One. 2012 Mar 14;7(3):e31918. doi: 10.1371/journal.pone.0031918 (PMC3303470; doi:10.1371/journal.pone.0031918)

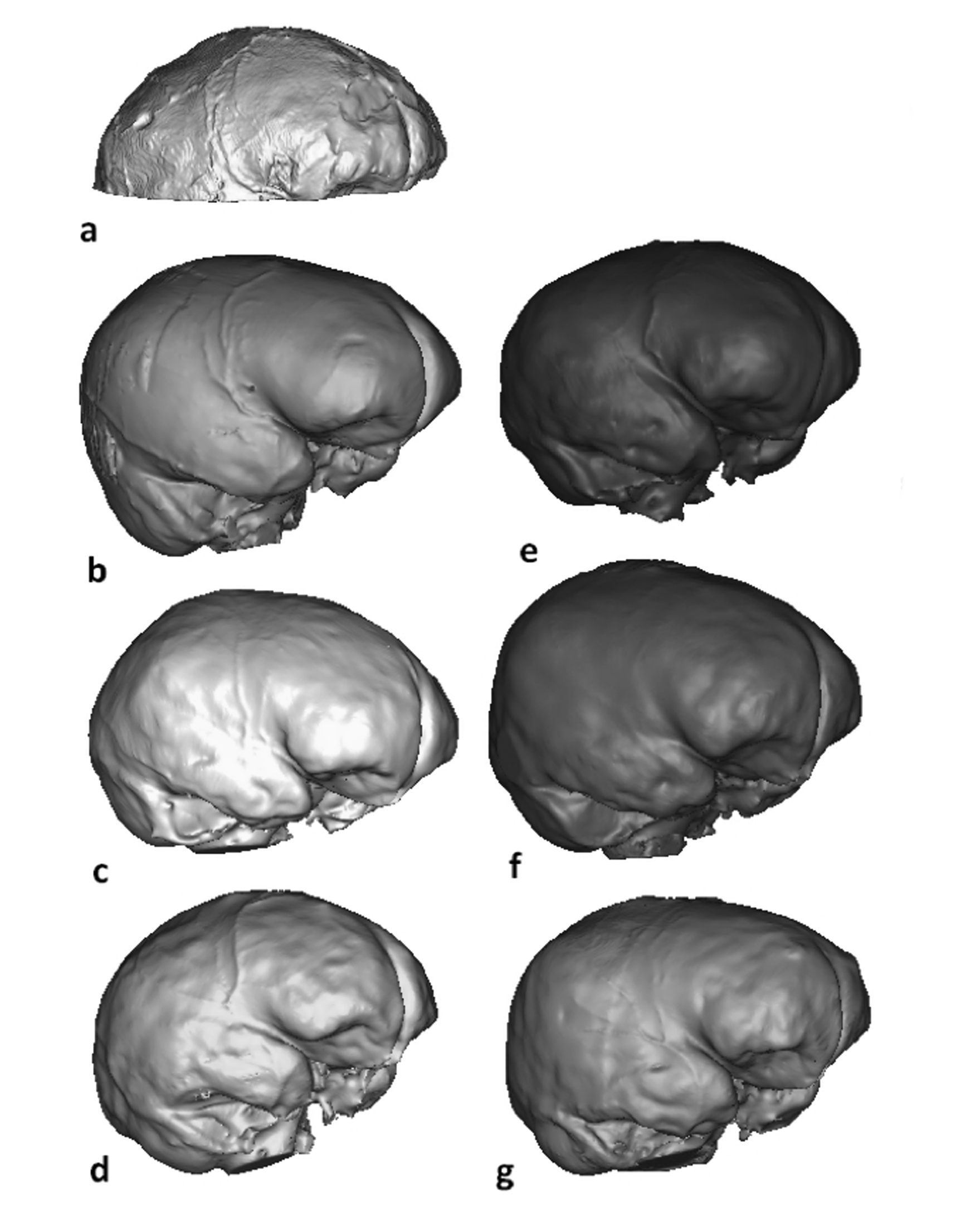

Supplement: Figure S1 — Endocasts generated from computed tomography. a, MLDG 1704, and San crania: b, NMB 4. c, NMB 1271. d, NMB 1640. e, NMB 1204. f, NMB 1707. g, NMB 1240. (TIF) [file pone.0031918.s001.tif]

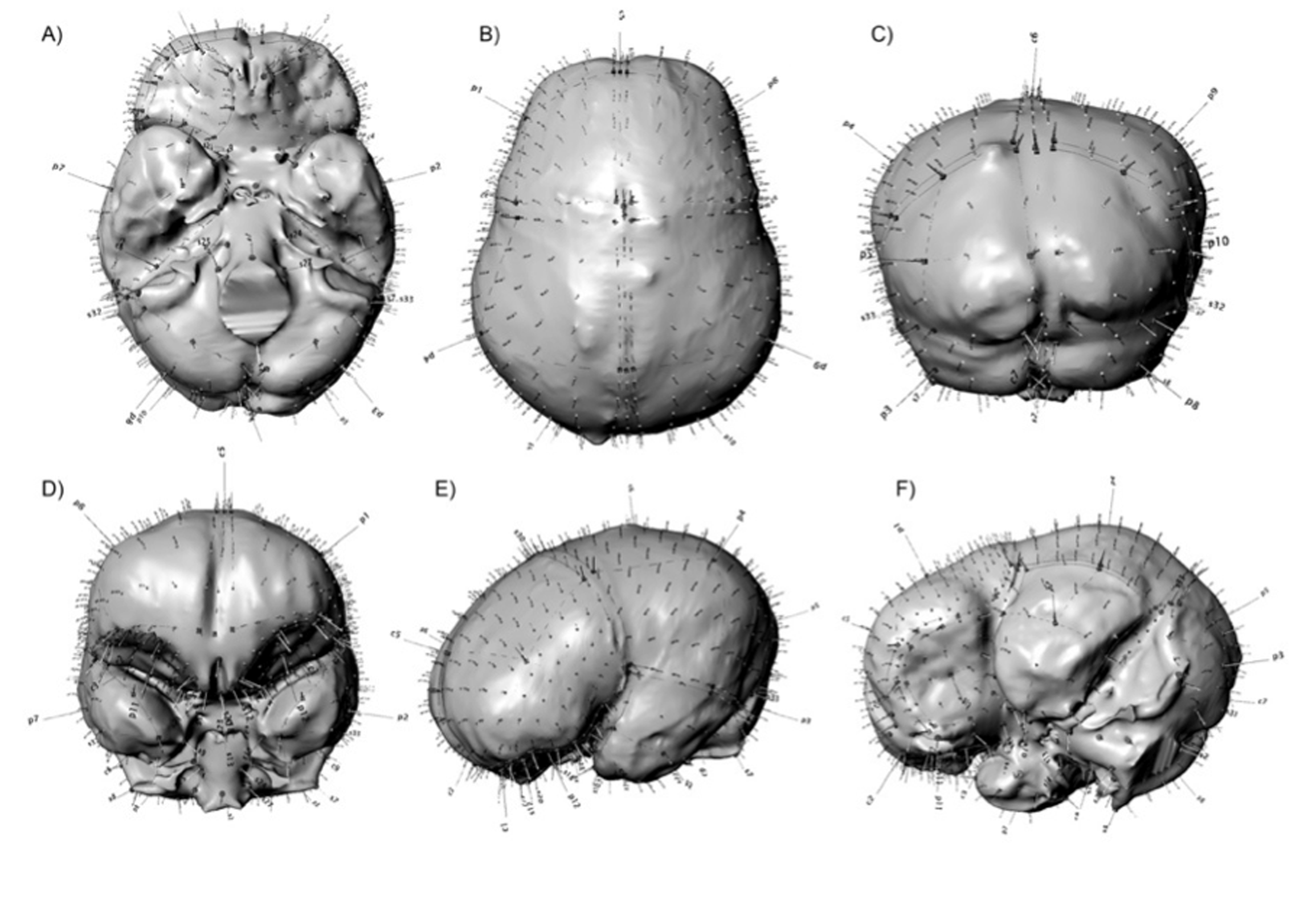

Supplement: Figure S2 — Varying views of the BW 1204 modern human (San) endocranium specimen showing the landmark and slid semilandmark template as applied to each of the 6 modern human specimens. Aspect viewed: A) inferior, B) superior, C) frontal, D) ¾ frontal-inferior E) lateral F) ¾ inferior-lateral. (TIF) [file pone.0031918.s002.tif]

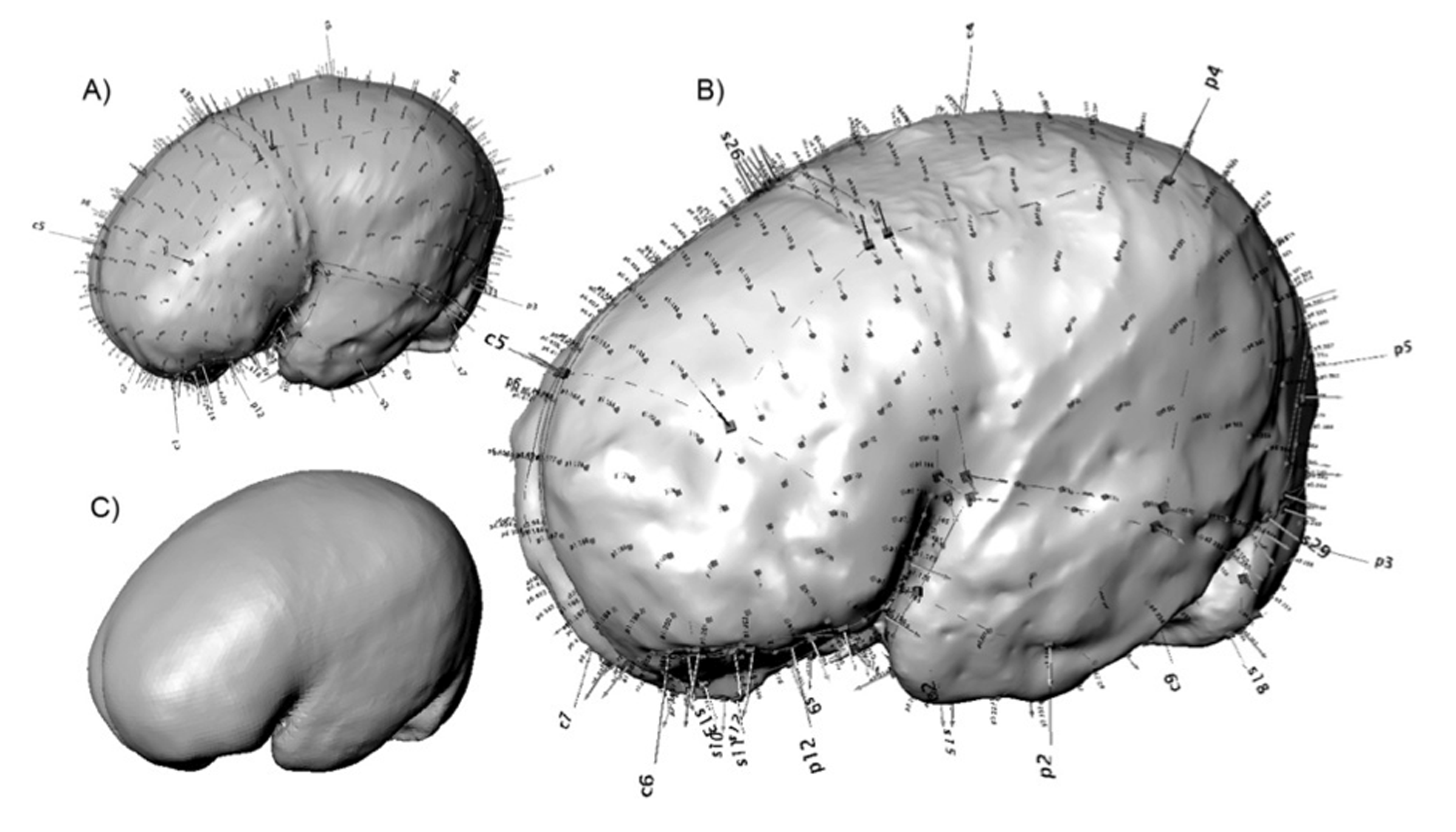

Supplement: Figure S3 — Landmark template. A) applied to BW 1204 (as in Figure S2), B) same landmark template applied to specimen BW 1240, C) and mean modern (San) endocranial shape generated from the average landmark configuration of the 6 modern human specimens. (TIF) [file pone.0031918.s003.tif]

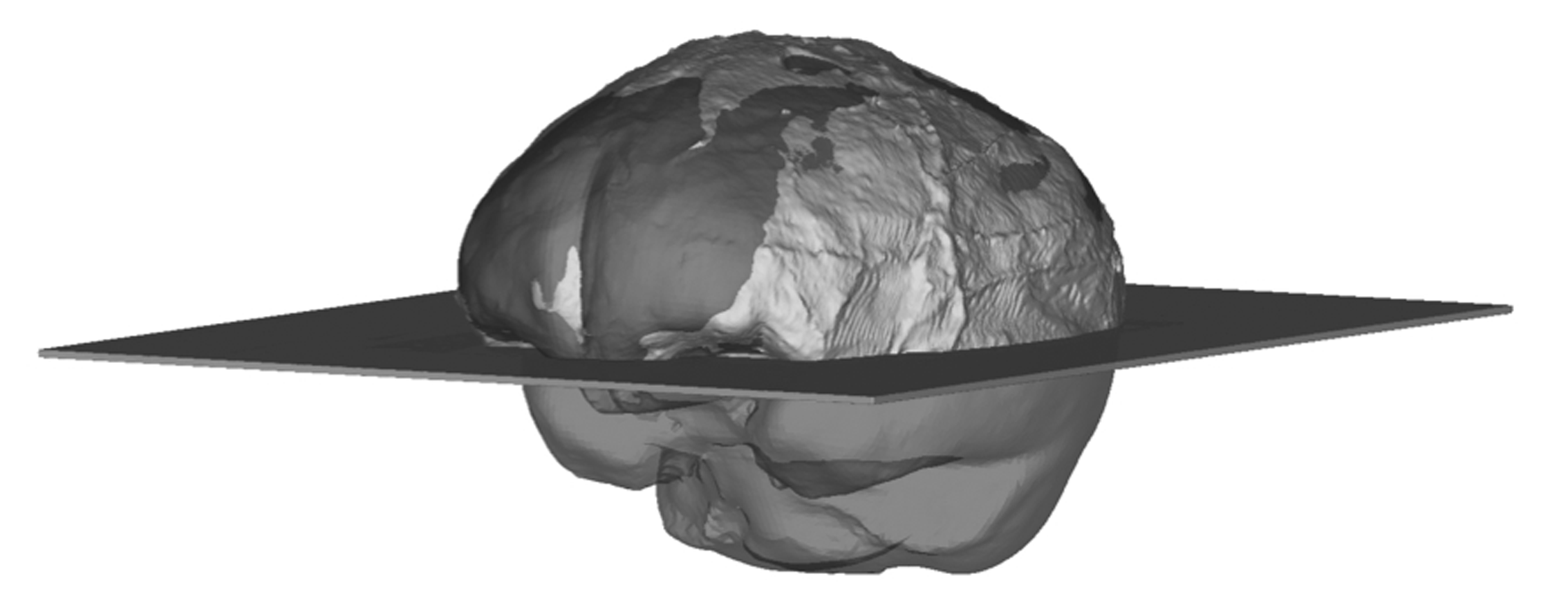

Supplement: Figure S4 — Registered mean San (dark gray) and MLDG 1704 (light gray) endocasts superimposed showing cutting plane. (TIF) [file pone.0031918.s004.tif]
