# Supplementary material for: Human Remains from the Pleistocene-Holocene Transition of Southwest China Suggest a Complex Evolutionary History for East Asians
Source: PLoS One. 2012 Mar 14;7(3):e31918. doi: 10.1371/journal.pone.0031918 (PMC3303470; doi:10.1371/journal.pone.0031918)
Supplement: Table S1 — Radiocarbon data for Maludong. (DOCX) [file pone.0031918.s005.docx]

Table S1. Radiocarbon data for Maludong.

| Lab ID | Carbon mass | Carbon mass | Mass used for | Carbon mass | Carbon mass | Fe | del-^13^C | del-^13^C |
| --- | --- | --- | --- | --- | --- | --- | --- | --- |
|  | Before pretreatment | After pretreatment | Combustion | value | error |  |  |  |
|  | (mg) | (mg) | (mg) | (mg) | (mg) | (mg) | per mil | error |
| OZM143 | 11.7 | 7.6 | 3.59 | 2.47 | 0.03 | 1.97 | -24.7 | 0.1 |
| OZM144 | 13.8 | 7.0 | 4.02 | 2.68 | 0.04 | 2.19 | -25.6 | 0.3 |
| OZM145 | 17.6 | 12.6 | 3.77 | 2.65 | 0.04 | 2.11 | -27.6 | 0.1 |
| OZM146 | 34.4 | 19.2 | 3.69 | 2.58 | 0.04 | 2.48 | -24.8 | 0.1 |
| OZM147 | 9.4 | 0.40 | 0.40 | 0.35 | 0.01 | 0.79 | -21.8 | 0.4 |
| OZM148 | 14.6 | 12.0 | 4.23 | 2.94 | 0.04 | 2.41 | -25.3 | 0.1 |
| OZM149 | 14.6 | 10.3 | 3.92 | 3.14 | 0.04 | 2.65 | -25.5 | 0.1 |
| OZM150 | 19.8 | 8.6 | 2.80 | 1.87 | 0.03 | 2.01 | -24.4 | 0.1 |
| OZM151 | 31.4 | 7.4 | 3.32 | 2.19 | 0.03 | 2.35 | -24.0 | 0.1 |
| OZM152 | 33.1 | 3.8 | 2.17 | 1.41 | 0.02 | 1.71 | -24.7 | 0.1 |
| OZM153 | 27.7 | 11.4 | 3.01 | 2.02 | 0.03 | 2.17 | -24.1 | 0.1 |
| OZM154 | 16.4 | 9.2 | 3.78 | 2.44 | 0.03 | 2.41 | -25.3 | 0.1 |
| OZM155 | 36.4 | 31.8 | 5.12 | 3.15 | 0.04 | 2.44 | -27.1 | 0.4 |
| OZM369 | 29.5 | 3.5 | 2.48 | 1.71 | 0.02 | 1.64 | -25.6 | 0.1 |
| OZM870 | 35.8 | 21.6 | 4.41 | 2.96 | 0.04 | 1.74 | -25.0 | 0.1 |
