# Supplementary material for: Human Remains from the Pleistocene-Holocene Transition of Southwest China Suggest a Complex Evolutionary History for East Asians
Source: PLoS One. 2012 Mar 14;7(3):e31918. doi: 10.1371/journal.pone.0031918 (PMC3303470; doi:10.1371/journal.pone.0031918)
Supplement: Text S1 — Archaeomagnetics - results. (DOCX) [file pone.0031918.s006.docx]

Text S1 – Archaeomagnetics – Results

The Maludong sediments have very high magnetic susceptibility values (χ_LF_ up to 12.3 x 10^-6^ m^3^ kg^-1^ and mean 9.6 x 10^-6^ m^3^ kg^-1^) considering the fact that they were formed on a karst landscape. Extremely high frequency dependence of magnetic susceptibility (χ_FD_%) values occurs between 14 and 8% (mean 11.3%) and is due to grains sitting across the superparamagnetic (SP) to single domain (SD) grain size boundary and not due to low coercivity multi-domain (MD) grains as shown by a lack of a MD peak in low temperature magnetic susceptibility curves. These χ_FD_% values are close to the maximum predicted value of natural samples for this grain size with χ_FD_% values >10%, suggesting that such grain sizes make up 75% of the assemblage [1]. The mineralogy of the bulk samples is extremely similar up through the section at Maludong. All layers are dominated by fine to ultra-fine grained low coercivity magnetite as shown by Curie temperatures of ~575^o^C, high χ_FD_% values (14-8%) and complete saturation of IRM by 100mT and a S-ratio of 1. The samples have undergone a high degree of ferromagnetic enhancement before they were deposited in the cave. This degree of ferromagnetic enhancement is consistent with a high rainfall, warm climatic regime and is consistent with formation in the Bølling-Allerød interstadial, consistent with data from China [2].

References

1. Brown KS, Marean CW, Herries AIR, Jacobs Z, Tribolo C et al. (2009) Fire as an engineering tool of early modern humans. Science 325: 859-862.
2. Kramer A, Herzschuh U, Mischke S, Zhang C (2010) Late glacial vegetation and climate oscillations on the southeastern Tibetan Plateau inferred from the Lake Naleng pollen profile. Quat Res 73: 324-335.
