# Supplementary material for: Human Remains from the Pleistocene-Holocene Transition of Southwest China Suggest a Complex Evolutionary History for East Asians
Source: PLoS One. 2012 Mar 14;7(3):e31918. doi: 10.1371/journal.pone.0031918 (PMC3303470; doi:10.1371/journal.pone.0031918)
Supplement: Text S2 — Additional references: sources of metrical and morphological data and dating estimates. (DOCX) [file pone.0031918.s007.docx]

Text S2 – Additional refereces: sources of metrical and morphological data and dating estimates

1. Ahern JCM, Karavanic I, Paunovic M, Jankovic I, Smith FH (2004). New discoveries and interpretations of hominid fossils and artifacts from Vindija Cave, Croatia. J Hum Evol 46: 27-67.
2. Baba H, Shuichiro HN, Ohyama, S (1998). Minatogawa hominid fossils and the evolution of late Pleistocene humans in East Asia. Anthropol Sci 106 (Supplement): 27-45.
3. Brown, P (2011). Minatogawa 1. Available: http://www-personal.une.edu.au/~pbrown3/Minat.html via the internet. Accessed 5 Dec 2011.
4. Cuong NL (1986). Two early Hoabinhian crania from Thanh Hoa Province, Vietnam. Z Morph Anthropol 77: 11-17.
5. Crevecoeur I, Trinkaus, E (2004). From the Nile to the Danube: a comparison of the Nazlet Khater 2 and Oase 1 early modern human mandibles. Anthropologie 152: 203-213.
6. Crevecoeur I, Rougier H, Grine F, Froment A (2009). Modern human cranial diversity in the late Pleistocene of Africa and Eurasia: evidence From Nazlet Khater, Pestera cu Oase, and Hofmeyr. Am J Phys Anthropol 140: 347-358.
7. Day MH, Stringer CB (1982). Les restes crâniens D’Omo-Kibish et leur classification à l’interieur du genre Homo. In: de Lumley H, editor. Homo erectus *et la Place de l'Homme et Tautavel Parmi les Hominidés Fossiles*, Nice, Prétirage, pp. 814-846.
8. Frayer DW, Jelinek J, Oliva M, Wolpoff MH (2006). Aurignacian male crania, jaws and teeth from the Mladec Caves, Moravia, Czech Republic. In: Teschler-Nicola M, editor. *Early modern humans at the Moravian Gate: Mladec Caves and their remains* (ed M. Teschler-Nicola), New York, Springer-Wien, pp. 185-272.
9. Formicola V, Pettitt PB, Del Lucchese AA (2004). Direct AMS radiocarbon date on the Barma Grande 6 Upper Paleolithic skeleton. Curr Anthropol 45: 114-118.
10. Grine FE, Pearson OM, Klein RG, Rightmire GP (1998). Additional human fossils from Klasies River Mouth, South Africa. J Hum Evol 35: 95-107.
11. Grine FE, Gunz P, Betti-Nash L, Neubauer S, Morris AG (2010). Reconstruction of the late Pleistocene human skull from Hofmeyr, South Africa. J Hum Evol 59: 1-15.
12. Hanihara T (2006). Interpretation of craniofacial variation and diversification of East and Southeast Asians. In: Oxenham M, Tayles N, editors. *Bioarchaeology of Southeast Asia*, Melbourne, Cambridge University Press, pp. 99-111.
13. Holloway R. (1981). Volumetric and asymmetry determinations on recent hominid endocasts: Spy I and II, Djebel Irhoud I, and the Sale *Homo erectus* specimens, with some notes about Neandertal brain size. Am J Phys Anthropol 55: 385-393.
14. Howells WW (1996). WW Howells’ craniometric data. Available: <http://konig.la.utk.edu/howells.htm>. Accessed 6 Dec 2011.
15. Indriati E, Swisher CC III, Lepre C, Quinn RL, Suriyanto RA et al. (2011). The Age of the 20 Meter Solo River Terrace, Java, Indonesia and the Survival of Homo erectus in Asia. PLoS One 6(6): e21562.
16. Kaifu Y, Aziz F, Baba H (2005). Hominid mandibular remains from Sangiran: 1952–1986 collection. Am J Phys Anthropol 128: 497-519.
17. Kaifu Y, Aziz F, Indriati E, Jacob T, Kurniawan I et al. (2008). Cranial morphology of Javanese *Homo erectus*: new evidence for continuous evolution, specialization, and terminal extinction. J Hum Evol 55: 551-580.
18. Kubo D, Kono RT (2011). Endocranial restoration and volume estimation of the Minatogawa IV cranium using micro-CT and 3D printer systems. Anthropol Sci 119: 203-209.
19. Kubo D, Kono RT, Suwa G (2011). A micro-CT based study of the endocranial morphology of the Minatogawa I cranium. Anthropol Sci 119: 123-135.
20. Liu W, Jin C-Z, Zhang Y-Q, Cai YJ, Xing S et al. (2010). Human remains from Zhirendong, South China, and modern human emergence in East Asia. Proc Natl Acad Sci 107: 19201-19206.
21. Liu W, Wu X, Pei S, Wu X, Norton CJ (2010). Huanglong Cave: A late Pleistocene human fossil site in Hubei Province, China. Quat Int 211: 29-41.
22. Matsu’ura S, Kondo, M (2011). Relative chronology of the Minatogawa and the Upper Minatogawa series of human remains from Okinawa Island, Japan. Anthropol Sci 119: 173-182.
23. Matsumura H (2006). The population history of Southeast Asia viewed from morphometric analyses of human skeletal and dental remains. In: Oxenham M, Tayles, N, editors. *Bioarchaeology of Southeast Asia*, Melbourne, Cambridge University Press, pp. 33-58.
24. Matsumura H, Pookajorn S (2005). A morphometric analysis of the late Pleistocene human skeleton from the Moh Khiew Cave in Thailand. Homo-J Comp Hum Biol 56: 93-118.
25. Matsumura H, Zuraina M (1999). Metric Analyses of an early Holocene human skeleton from Gua Gunung Runtuh, Malaysia. Am J Phys Anthropol 109: 327-340.
26. Matsumura H, Yoneda M, Dodo Y, Oxenham MF, Cuong NL et al*.* (2008). Terminal Pleistocene human skeleton from Hang Cho Cave, northern Vietnam: implications for the biological affinities of Hoabinhian people. Anthropol Sci 116: 201-217.
27. McCown TD, Keith A (1939). *The Stone Age of Mount Carmel: the Fossil Human Remains from the Levalloiso-Mousterian*. Vol. 2, Oxford, Oxford at the Clarendon Press.
28. McDougall I, Brown FH, Fleagle JG (2005). Stratigraphic placement and age of modern humans from Kibish, Ethiopia. Nature 433: 733-736.
29. Millard AR (2008). A critique of the chronometric evidence for hominid fossils: I. Africa and the Near East 500-50 ka. J Hum Evol 54: 848-874.
30. Pietrusewsky M (2006). A multivariate craniometric study of prehistoric and modern inhabitants of Southeast Asia, East Asia and surrounding regions: a human kaleidoscope? In: Oxenham M, Tayles N, editors. *Bioarchaeology of Southeast Asia*, Melbourne, Cambridge University Press, pp. 59-90.
31. Shang H, Tong H, Zhang S, Chen F, Trinkaus E (2007). An early modern human from Tianyuan Cave, Zhoukoudian, China. Proc Natl Acad Sci 104: 6573-6578.
32. Shen G, Gao X, Gao B, Granger D (2009). Age of Zhoukoudian *Homo erectus* determined with ^26^Al/^10^Be burial dating. Nature 458: 198–200.
33. Simmons T, Falsetti AB, Smith FH (1991). Frontal bone morphometrics of southwest Asian Pleistocene hominids. J Hum Evol 20: 249-269.
34. Sládek V, Trinkaus E, Stefcakova A, Halouzka R (2002). Morphological affinities of the Sal’a 1 frontal bone. J Hum Evol 43: 787-815.
35. Smith FH, Ranyard GC (1980). Evolution of the supraorbital region in upper Pleistocene fossil hominids from south-central Europe. Am J Phys Anthropol 53: 589-610.
36. Smith FH, Ahern JC (1994). Additional cranial remains from Vindija Cave, Croatia. Am J Phys Anthropol 93: 275-280.
37. Stewart TD (1977). The Neanderthal skeletal remains from Shanidar Cave, Iraq: a summary of findings to date. Proc Am Phil Soc 121: 121-165.
38. Swisher CC III, Curtis GH, Jacob J, Getty AG, Suprijo A et al. (1994). Age of the earliest known hominids in Java, Indonesia. Science 263: 1118-1121.
39. Trinkaus E (1973). A reconsideration of the Fontechevade fossils. Am J Phys Anthropol 39: 25-35.
40. Trinkaus E (1987). The Neandertal face: evolutionary and functional perspectives on a recent hominid. J Hum Evol 16: 429-443.
41. Trinkaus E (2005). Early modern humans. Annu Rev Anthropol 34: 207-230.
42. Trinkaus E (2006). Modern human versus Neandertal evolutionary distinctiveness. Curr Anthropol 47: 597-620.
43. Trinkaus E, Arter DD, Franciscus RG (1996). A reconstruction of the Shanidar 4 facial skeleton. Paléorient 22: 51-66.
44. Trinkaus E, Milota S, Rodrigo R, Mircea G, Moldovan O (2003). Early modern human remains from the Pestera cu Oase, Romania. J Hum Evol 45: 245-253.
45. Trinkaus E, Biglari F, Mashkour M, Monchot H, Reyss J-L, et al. (2008). Late Pleistocene human remains from Welzmeh Cave, Western Iran. Am J Phys Anthropol 135: 371-378.
46. Vandermeesch B (1981). *Les Hommes Fossiles De Qafzeh (Israël). Cahiers de Paléontologie (Paléoanthropologie)*. Paris: Éditions du Centre National de la Recherche Scientifique.
47. Vallois MV, Vandermeersch B (1975). The Mousterian skull of Qafzeh (Homo VI). An anthropological study. J Hum Evol 4: 445-455.
48. Valladas H, Mercier N, Joron JL, McPherron SP, Dibble H et al. (2003). TL dates for the Middle Paleolithic site of Combe-Capelle Bas, France. J Archaeol Sci 30: 1443-1450.
49. Weidenreich F (1936). The mandibles of *Sinanthropus pekinensis*: a comparative study. Pal. Sinica. Vol. 7, Series D.
50. Weidenreich F (1936). The dentition of *Sinanthropus pekinensis*: a comparative odontography of the hominids. Pal Sinica Wholse Series No. 101, New Sereis D No. 1.
51. Weidenreich F (1945). The Keilor skull: a Wadjak type from southeast Australia. Am J Phys Anthropol 3:21-32.
52. White TD, Asfaw B, DeGusta D, Gilbert H, Richards GD et al. (2003). Pleistocene *Homo sapiens* from Middle Awash, Ethiopia. Nature 423: 742-747.
53. Wood BA (1991). Hominid Cranial Remains. In: *Koobi Fora Research Project*, vol. 4. Oxford, Clarendon Press.
54. Wolpoff MH, Frayer DW, Jelínek J (2006). Aurignacian female crania and teeth from the Mladec Caves, Moravian Czech Republic. In *Early modern humans at the Moravian Gate: Mladec Caves and their remains* (ed M. Teschler-Nicola), pp. 272-340. New York, Springer-Wien.
55. Wu X, Poirier FE (1995). *Human Evolution in China*. Oxford, Oxford University Press.
56. Wu X, Liu W, Gao X, Yin G (2006). Huanglong Cave, a new late Pleistocene hominid site in Hubei Province, China. Chin Sci Bull 51: 2493-2499.
57. Wu X, Schepartz L, Wu L (2010). A new *Homo erectus* (Zhoukoudian V) brain endocast from China. Proc. R. Soc. B 277: 337-344.
58. Wu X, Liu W, Dong W, Que J-M, Wang YF (2008). The brain morphology of *Homo* Liujiang cranium fossil by three-dimensional computed tomography. Chin Sci Bull 53: 2513-2519.
59. Wu X, Holloway RL, Schepartz LA, Song X (2011). A new brain endocast of *Homo erectus* from Hulu Cave, Nanjing, China. Am J Phys Anthropol 145: 452-460.
